# From In Vitro Screening to In Vivo Evaluation of Essential-Oil-Based Formulation Against Avian Pathogenic *Escherichia coli*

**DOI:** 10.3390/ani16193099

**Published:** 2026-10-02

**Authors:** Milenko Rikić, Zoran Ružić, Marko Pajić, Dalibor Todorović, Slobodan Knežević, Nebojša Kladar, Zorana Kovačević

**Affiliations:** 1 Scientific Veterinary Institute Novi Sad, Rumenački put 20, 21000 Novi Sad, Serbia; milenko.r@niv.ns.ac.rs (M.R.); markopajic@niv.ns.ac.rs (M.P.); dalibor@niv.ns.ac.rs (D.T.); slobodan.knezevic@niv.ns.ac.rs (S.K.); 2 Department of Veterinary Medicine, Faculty of Agriculture, University of Novi Sad, Trg Dositeja Obradovića 8, 21000 Novi Sad, Serbia; zorana.kovacevic@polj.edu.rs; 3 Center for Medical and Pharmaceutical Investigations and Quality Control, Faculty of Medicine, University of Novi Sad, Hajduk Veljkova 3, 21000 Novi Sad, Serbia; nebojsa.kladar@mf.uns.ac.rs; 4 Department of Pharmacy, Faculty of Medicine, University of Novi Sad, Hajduk Veljkova 3, 21000 Novi Sad, Serbia

**Keywords:** avian pathogenic *Escherichia coli*, antimicrobial resistance, essential oils, oregano, thyme, antimicrobial stewardship

## Abstract

Antimicrobial resistance in avian pathogenic *Escherichia coli* (APEC) poses a significant challenge to poultry production, highlighting the need for effective alternatives to conventional antimicrobials. This study aimed to evaluate the antibacterial activity of selected essential oils (EOs) against multidrug-resistant APEC isolates and assess the in vivo effects of an oregano–thyme EO-based formulation in experimentally infected broilers. Six EOs were screened in vitro using the broth microdilution method, and oregano and thyme EOs, showing the strongest activity (MIC 0.003% and 0.003–0.007%, respectively), were further evaluated in two combinations. The more active combination was formulated for administration via drinking water and tested in 420 Ross 308 broilers experimentally challenged with APEC. Birds were allocated to untreated control, infected untreated, EO-treated, enrofloxacin-treated, and enrofloxacin + reduced-dose EO formulation groups. Mortality did not differ significantly among groups. A binomial GLMM identified significant differences in faecal consistency among experimental groups (likelihood-ratio χ^2^ = 32.90, df = 4, *p* < 0.001), while no significant differences were observed in body weight, feed intake, or overall FCR. Significant differences were detected in AST, glucose, total bilirubin, and calcium concentrations. Overall, the EO-based formulation demonstrated antibacterial activity in vitro and was associated with changes in intestinal status following APEC challenge, while no consistent improvement in productive performance was observed. These findings support further investigation of EO-based formulations as potential non-antibiotic approaches for managing APEC-associated intestinal disturbances in broiler chickens.

## Simple Summary

Avian pathogenic *Escherichia coli* (APEC) is an important cause of colibacillosis in poultry, while increasing antimicrobial resistance highlights the need for alternative approaches to disease control. Essential oils have attracted interest due to their potential antibacterial properties and possible application in poultry production. In this study, several essential oils were screened against APEC isolates, and a formulation based on the most active oils was further evaluated in experimentally infected broilers. The study provides preliminary evidence on the potential of an essential-oil-based approach as a non-antibiotic option for the control of avian colibacillosis and supports further investigation under practical production conditions.

## 1. Introduction

Avian colibacillosis is one of the most significant bacterial diseases affecting global poultry production, causing substantial economic losses and compromising animal health and welfare [1], which is caused by avian pathogenic *Escherichia coli* (APEC), a subgroup of extraintestinal pathogenic *E. coli* (EExPEC) capable of inducing a broad spectrum of localized and systemic infections in poultry [2]. Its clinical presentation ranges from sudden death caused by peracute septicaemia to subacute and chronic forms characterized by fibrinopurulent lesions, most notably airsacculitis, pericarditis, and perihepatitis [3,4]. The economic consequences include increased mortality, impaired growth performance, reduced feed conversion efficiency, decreased egg production, and carcass condemnation at slaughterhouses [1,3]. Traditionally, the control of colibacillosis has relied substantially on antimicrobial therapy [4,5]. However, the extensive and inappropriate use of antimicrobials has played a major role in the emergence and global dissemination of antimicrobial resistance (AMR) among APEC isolates, raising serious concerns for both veterinary and public health [5,6]. The spread of resistant bacteria through the food chain, environmental contamination, and direct contact pathways contributes to the global burden of AMR, highlighting the urgent need for development of sustainable and effective alternative strategies in poultry production. These concerns, together with increasing restrictions on antimicrobial use in food-producing animals, emphasize the need to develop effective and sustainable strategies that can reduce dependence on conventional antimicrobial therapy [7].

Plant-derived bioactive compounds, particularly essential oils (EOs), have consequently received increasing attention as potential tools for controlling bacterial diseases in poultry [8]. EOs are complex mixtures of volatile plant compounds, rich in phenolics and terpenoids such as carvacrol, thymol, eugenol, and cinnamaldehyde, exhibiting antibacterial, antioxidant, anti-inflammatory, and immunomodulatory properties [9]. Their antibacterial activity, confirmed in vitro against *E. coli* and APEC strains, is primarily mediated through disruption of bacterial membranes, metabolic interference, and inhibition of virulence factors [10]. EOs rich in thymol and carvacrol, particularly those derived from *Thymus vulgaris*, *Thymus serpyllum*, and *Origanum vulgare*, exhibit strong antibacterial activity against major veterinary pathogens, supporting their potential application in livestock production [11].

In addition to their direct antimicrobial effects, EO supplementation has been associated with improved zootechnical performance and feed conversion, modulates intestinal microbiota by suppressing pathogens and promoting beneficial bacteria, and enhances immune responses and oxidative status, thereby contributing to overall health and disease resistance in poultry [12,13]. EO and other plant-derived bioactive compound supplementation has been shown to modulate gut microbiota composition, enhance microbial diversity and community stability, and maintain microbial balance in poultry under pathogen challenge, whereas antibiotic treatment has been associated with early disruption of microbiota architecture and induction of dysbiosis [14]. Despite these promising findings, the practical implementation of EO-based pharmaceutical formulation on commercial farms remains limited, primarily due to their relatively high cost, the lack of standardized formulations, and insufficient data from field trials [7,10].

Therefore, the present study aimed to develop and evaluate EO-based formulation suitable for administration via drinking water as a potential strategy of colibacillosis in broiler chickens. Finally, in vivo assessment was performed by evaluation of impact on clinical condition and production performance parameters after EO-based formulation treatment. This approach was designed to determine whether in vitro antimicrobial activity could be translated into measurable health and production benefits under in vivo conditions. In this context, the significance of the present study lies in exploring an essential-oil-based formulation as a potential non-antibiotic approach for APEC-associated disease, with the broader aim of reducing reliance on conventional antibiotics and contributing to efforts to mitigate antimicrobial resistance in poultry production.

## 2. Materials and Methods

### 2.1. Essential Oils and Chemical Analysis

The essential oils (EOs) used in this study were purchased in 2026 from eTERRA, Novi Sad, Serbia, and included oregano (*Origanum vulgare*, Lamiaceae), thyme (*Thymus vulgaris*, Lamiaceae), cinnamon (*Cinnamomum verum*, Lauraceae), rosemary (*Rosmarinus officinalis*, Lamiaceae), lavender (*Lavandula angustifolia*, Lamiaceae) and lemon (*Citrus limon*, Rutaceae) EOs. The semiquantitative profiling of essential oils was performed by gas chromatography tandem mass spectrometry (GC–MS) instrumental method (7890B GC System coupled to a 5977A MSD; Agilent Technologies, Waldbronn, Germany). Briefly, 20 µL of the EO was diluted in 980 µL of n-hexane and injected by setting inlet temperature at 220 °C and split mode 20:1. The compounds were separated on an HP-5MS column (30 m × 0.250 mm × 0.25 μm; Agilent Technologies) with constant helium flow (1 mL/min) and according to the following temperature programme: initial oven temperature was 60 °C, the temperature was increased to 246 °C at a rate of 3 °C/min, maintained at 246 °C for 3 min, further raised to 280 °C, and maintained for 5 min. The MS spectra were obtained in scan mode (*m*/*z* = 30–550), while the transfer line temperature was set at 285 °C. The identification of EO compounds was performed by analysing relative retention indices (RI) and mass spectra available in the NIST v14 spectral library [15].

### 2.2. In Vitro Antimicrobial Activity of Essential Oils and Essential Oil-Based Formulation Against Avian Pathogenic E. coli Isolates

#### 2.2.1. Selection of Essential Oils for the Formulation

To evaluate the antibacterial effects of EOs on the growth of *E. coli,* 100% concentrated oregano, thyme, cinnamon, rosemary, lavender, and lemon EOs (eTERRA, Novi Sad, Serbia) were used. Seven *E. coli* isolates exhibiting a multidrug-resistant (MDR) phenotype, defined as non-susceptibility to at least one antimicrobial agent in three or more antimicrobial categories [16], were included in the study. The isolates were recovered from faecal swabs collected from seven broiler farms in the Autonomous Province of Vojvodina, Republic of Serbia, and originated from the collection of bacterial isolates maintained at the Department of Clinical Bacteriology, Mycology and Parasitology, Scientific Veterinary Institute “Novi Sad”, Novi Sad, Serbia. *E. coli* isolates had previously been molecularly characterized by PCR for the presence of virulence genes according to the criteria described by Johnson et al. (2008) [17] and Johnson et al. (2010) [18] whereby the presence of two or more virulence genes was considered indicative of an avian pathogenic *E. coli* (APEC) phenotype. All seven *E. coli* isolates were classified as APEC (Supplementary Table S1). The antibacterial activity of the tested EOs was evaluated based on their ability to inhibit the growth of the selected MDR-APEC *E. coli* isolates.

The inhibitory effects of the EOs on the growth of APEC isolates were evaluated using the broth microdilution method, according to the principles of the standardized procedure described in CLSI M07-Ed12 [19]. Acetone (≥99.5%; Sigma-Aldrich, St. Louis, MO, USA) was used as the solvent for the preparation of the initial essential oil solutions. The initial concentration of each essential oil was 1%, followed by two-fold serial dilutions in Mueller-Hinton broth (Oxoid Ltd., Basingstoke, UK; Cat. No. CM0405B) in flat-bottom microtiter plates (Sarstedt AG & Co. KG, Nümbrecht, Germany). The minimum inhibitory concentration (MIC) was defined as the lowest concentration of the EO that resulted in complete inhibition of visible growth of the tested APEC isolate. Appropriate bacterial growth, medium sterility, and solvent controls were included for each isolate to exclude any potential inhibitory effect of acetone.

#### 2.2.2. Inhibitory Effect of Two Oregano–Thyme Essential Oil Combinations

EOs that show the strongest antibacterial activity against the selected MDR-APEC isolates are used for further testing in the combination. Two different oregano–thyme EO combinations were evaluated for antibacterial activity against the selected MDR-APEC isolates. Combination 1 contained oregano and thyme EOs at a ratio of 30:70 (*v*/*v*), whereas Combination 2 contained the same EOs at a ratio of 46.15:53.85 (*v*/*v*). For MIC determination, the initial test solution of Combination 1 was prepared by combining 76.8 µL of oregano EO and 179.2 µL of thyme EO and adjusting the final volume to 20 mL with acetone. The initial test solution of Combination 2 was prepared by combining 153.8 µL of oregano EO and 179.2 µL of thyme EO and adjusting the final volume to 20 mL with acetone.

The inhibitory effect of the oregano–thyme essential oil formulation on the growth of APEC isolates was evaluated using the broth microdilution method, according to the principles of the standardized procedure described in CLSI M07-Ed12. The formulation contained oregano essential oil at a proportion of 43.82% and thyme essential oil at a proportion of 56.18%. Acetone (≥99.5%; Sigma-Aldrich, St. Louis, MO, USA) was used as the solvent because of the hydrophobic nature of essential oils and their limited solubility in aqueous Mueller-Hinton broth (Oxoid Ltd., Basingstoke, UK; Cat. No. CM0405B). The microdilution assay was performed in flat-bottom microtiter plates (Sarstedt AG & Co. KG, Nümbrecht, Germany) using two-fold serial dilutions of the oregano–thyme formulation in Mueller-Hinton broth. The minimum inhibitory concentration (MIC) was defined as the lowest concentration of the oregano–thyme formulation that resulted in complete inhibition of visible growth of the tested APEC isolate. Appropriate bacterial growth, medium sterility, and solvent controls were included for each isolate to exclude any potential inhibitory effect of acetone.

### 2.3. Development and Chemical Characterization of Essential Oil-Based Formulation

The EO-based formulation used in study performed on broilers was prepared as concentrated nanoemulsion according to the previously described spontaneous emulsification procedure [20]. The in vitro antimicrobial potential determined for essential oils used in this research suggested the prominent activity of oregano and thyme EOs. Therefore, 30 g of oregano and 35 g of thyme essential oils were initially mixed with 130 g of polysorbate 20 (used as surfactant), after which the obtained mixture was added at a rate of 0.2 mL/min to 805 g of double-distilled water (at temperature of 25 °C and by using continuous magnetic stirring (600 rpm)). The stability of the obtained emulsion was evaluated by analysis of droplet size (AxioLab A1 microscope equipped with camera Axiocam ERc 5s, Carl Zeiss Microscopy GmbH, Jena, Germany) and polydispersity index (PDI), initially after preparation, and after 7 days of storage at 25 °C, which provided satisfactory stability parameters. Further, the obtained preparation was analysed for thymol and carvacrol quantities, since these compounds have been previously recognized for their strong antimicrobial potential. Briefly, 1 g of emulsion was extracted with 5 mL of internal standard solution (ketamine in diethyl ether, c = 0.5 µg/mL), and further analysed according to the previously developed and validated analytical method [21]. For administration to broilers, the concentrated formulation was added to drinking water at a concentration of 1 mL/L, it was diluted 1000 times.

### 2.4. Animals and Experimental Design

The in vivo trial was conducted over a period of 34 days using 420 one-day-old Ross 308 broiler chicks of mixed sex. Birds were randomly allocated to 20 floor pens, with 21 birds per pen, and assigned into five experimental groups with four replicate pens each (84 birds per group). Each pen contained an equal number of male and female birds. All pens were of equal dimensions, bedded with fresh wood-shaving litter, and maintained under uniform conditions with respect to lighting, ventilation. Water and a standard commercial broiler diet were provided ad libitum. Ambient temperature was adjusted according to standard Ross 308 broilers brooding recommendations. Birds had ad libitum access to drinking water and a commercial three-phase broiler diet manufactured by Biovita, Vajska, Serbia. The feeding programme consisted of PT1 starter diet during the first 7 days of the trial, PT2 grower diet during the intermediate period, and PT3 finisher diet during the final 7 days. Prior to the experiment, all three diets were subjected to chemical and microbiological analyses. The analysed crude protein contents were 24.30%, 22.15%, and 19.71% for PT1, PT2, and PT3, respectively; crude fat contents were 9.84%, 9.64%, and 10.74%; crude fibre contents were 4.38%, 4.50%, and 3.98%; calcium contents were 0.88%, 0.77%, and 0.68%; and phosphorus contents were 0.52%, 0.64%, and 0.38%, respectively. Microbiological examination showed total microbial counts of 31,000, 26,000, and 26,000 CFU/g and yeast and mould counts of 4000, 6000, and <100 CFU/g for PT1, PT2, and PT3, respectively. *Salmonella* spp. were not detected in 50 g of any diet sample, while *Clostridium perfringens* counts were <10 CFU/g in all three diets. All analysed parameters were within the corresponding reference limits. The five experimental groups, together with their corresponding treatments and infection status, are presented in Table 1.

The EO-based formulation was administered via drinking water at a concentration of 1 mL/L in Group III. Enrofloxacin oral solution (100 mg/mL; Enrocin-SP oral, VET-AGRO Multi Trade Company Sp. z o.o., Lublin, Poland) was selected based on the in vitro susceptibility of the challenge strain and administered at 10 mg/kg body weight per day in accordance with the manufacturer’s instructions. The daily volume of the commercial product was calculated separately for each pen based on the mean body weight and the number of live birds and ranged from approximately 2.6 to 2.9 mL per pen per day. In Group V, enrofloxacin was administered at the same dose of 10 mg/kg body weight per day, dissolved in a restricted volume of 1 L of drinking water per pen. The medicated water was consumed within approximately 4 h, after which the EO-based formulation was administered at a reduced concentration of 0.4 mL/L for the remainder of the day. Thus, Group V represented an enrofloxacin + reduced-dose EO formulation regimen rather than administration of both agents at their respective full-dose conditions. Medicated drinking water was prepared fresh daily and served as the sole source of drinking water throughout the five-day treatment period (days 20–24). Experimental infection with avian pathogenic *Escherichia coli* (APEC) was performed on day 15 of the trial. Birds assigned to Groups II–V each received 0.5 mL of a bacterial suspension containing 10^10^–10^11^ CFU/mL of the challenge strain, administered by oral gavage [22]. Birds in Group I received an equivalent volume of sterile physiological saline using the same administrative procedure. The interval between infection and the initiation of treatment was five days, during which clinical signs of infection, faecal consistency, and the general condition of each flock were monitored daily. By day 20, a noticeable alteration in faecal consistency was observed in the APEC-challenged groups, representing the first detectable clinical manifestation of the experimental challenge and the practical indication for initiating treatment.

The experimental protocol was approved by the Animal Ethics Committee of the Veterinary Directorate of the Ministry of Agriculture, Forestry and Water Management-Veterinary Directorate (Decision No. 000091154 2026, dated 14 January 2026).

### 2.5. Microbiological Analysis During In Vivo Trial

#### 2.5.1. Isolation and Identification of *E. coli* from Chickens Before and After Experimental Infection

Isolation of *E. coli* was performed from faecal swabs collected from one-day old chicks immediately after their placement in the experimental pens (day 0). The samples were initially incubated in Buffered Peptone Water (Oxoid Ltd., Basingstoke, UK; Cat. No. CM0509B) at 37 °C for 24 h and subsequently cultured on MacConkey Agar (Oxoid Ltd., Basingstoke, UK; Cat. No. CM0007B) at 37 °C for 24 h. Preliminary identification was based on colony morphology and lactose fermentation on MacConkey Agar. Final identification of *E. coli* isolates was performed using the automated biochemical identification system AUTOMIC-i600 (Autobio Diagnostics Co., Ltd., Zhengzhou, China). The same sampling, isolation, and identification procedures were applied on days 3, 5, and 19 after experimental infection.

#### 2.5.2. Antimicrobial Susceptibility Testing of *E. coli* Isolates Before and After Experimental Infection

Antimicrobial susceptibility testing of *E. coli* isolates was performed using the disk diffusion method in accordance with CLSI M02-Ed14 [23]. The following antibiotic discs at the indicated concentrations were used: ampicillin 10 µg (AMP), amoxicillin/clavulanic acid 20 + 10 µg (AMC), chloramphenicol 30 µg (CHL), ciprofloxacin 5 µg (CIP), gentamicin 10 µg (GEN), nalidixic acid 30 µg (NAL), streptomycin 10 µg (STR), sulfonamides 300 µg (SA), tetracycline 30 µg (TET), trimethoprim/sulfamethoxazole 1.25/23.75 µg (SXT), trimethoprim 5 µg (TMP), cefpodoxime 10 µg (CPD), cefotaxime 30 µg (CTX), and ceftazidime 30 µg (CAZ) (Bio-Rad, Marnes-la-Coquette, France).

#### 2.5.3. Molecular Characterization of APEC Isolates Based on Virulence Genes Before and After Experimental Infection

PCR detection of the virulence genes iroN, iutA, iss, hlyF, ompT, eitC, and cvaC, which are associated with the potential APEC phenotype according to Johnson et al. (2008) [17] and Johnson et al. (2010) [18], was performed on all *E. coli* isolates obtained on day 0 (before experimental infection) and on days 3, 5, and 19 after experimental infection. DNA extraction was performed by the boiling method using an overnight culture grown in Tryptone Soya Broth (Oxoid Ltd., Basingstoke, UK; Cat. No. CM0129B). Each PCR reaction was performed in a total volume of 25 µL and contained 12.5 µL of a commercially available TaqHotStart Master Mix (Qiagen, Hilden, Germany), 0.25 µL of each primer at a concentration of 10 µM, 7 µL of Milli-Q water, and 5 µL of extracted DNA. The PCR amplification conditions for each reaction were as described in the corresponding references provided in Supplementary Table S2. Electrophoresis was performed on a 2% agarose gel (Serva, Heidelberg, Germany) supplemented with 0.5% aqueous ethidium bromide solution (Serva, Heidelberg, Germany), using an ENDURO electrophoresis system (Labnet International, Edison, NJ, USA). The amplification products were visualized under UV light using a transilluminator (Vilber Lourmat, Collégien, France). The primer sequences, annealing temperatures, amplicon sizes, and corresponding references are presented in Supplementary Table S2.

#### 2.5.4. Preparation of the Bacterial APEC Inoculum for Experimental Infection of Chicks

The bacterial inoculum used for the experimental infection of chicks was prepared from an overnight culture (Tryptone Soya Broth) of the selected APEC isolate. For the in vivo challenge experiment, APEC isolate 2416 was selected as the challenge strain. Isolate 2416 exhibited a multidrug-resistant phenotype with resistance to ampicillin (AMP), chloramphenicol (CHL), ciprofloxacin (CIP), nalidixic acid (NAL), and tetracycline (TET), and carried the virulence-associated genes iroN, ompT, hlyF, iss, iutA, eitC, and cvaC. The inoculum was prepared from this characterized APEC isolate as described below. The bacterial suspension was initially adjusted to a 0.5 McFarland turbidity standard, corresponding to approximately 1.5 × 10^8^ CFU/mL for *E. coli*. The standardized bacterial suspension was subsequently concentrated by centrifugation, after which the supernatant was removed and the bacterial pellet was resuspended in sterile physiological saline (0.9% NaCl) to obtain a target bacterial concentration of approximately 10^10^–10^11^ CFU/mL. The final bacterial concentration was not independently confirmed by viable CFU enumeration. Each chick received 0.5 mL of the prepared bacterial suspension by oral administration.

### 2.6. Blood Sampling and Serum Biochemical Analyses

At the end of the trial (day 34), blood samples were collected from a total of 40 broilers, with eight birds sampled from each experimental group. Two birds were selected from each of the four replicate pens. Blood was collected by cardiac puncture into 9-mL serum tubes containing a clot activator (VACUSERA CAT Serum, Disera A.Ş., İzmir, Türkiye). Following clot formation, the samples were centrifuged at 8000 rpm for 5 min. The separated serum was refrigerated at 2–8 °C and analysed on the same day using an RT-1904C semi-automated chemistry analyser (Rayto Life and Analytical Sciences Co., Ltd., Shenzhen, China) and the corresponding commercial reagent kits according to the manufacturers’ instructions. The following serum biochemical parameters were determined: albumin, alanine aminotransferase (ALT), aspartate aminotransferase (AST), total bilirubin, cholesterol, creatinine, gamma-glutamyl transferase (GGT), glucose, calcium, magnesium, phosphorus, total protein, triglycerides and urea. Serum globulin concentration was calculated as the difference between total protein and albumin concentrations. Reference ranges applicable to this species were used for result interpretation.

### 2.7. In Vivo Assessment of Essential Oil-Based Formulation

Mortality was recorded daily in all pens. Each dead bird was weighed individually and subjected to gross post-mortem examination to document macroscopic lesions and determine the probable cause of death.

Faecal consistency was assessed daily at pen level using a binary scoring system (0 = normal faeces; 1 = watery or diarrhoeic faeces). Body weight was measured at pen level at weekly intervals on days 0, 7, 14, 21, 28 and 34 of the trial. Mean body weight per bird was calculated by dividing total pen weight by the number of birds present at each measurement point. Feed intake was recorded at pen level for each measurement interval ending on days 7, 14, 21, 28 and 34. Feed intake was calculated as the difference between the amount of feed supplied and the amount remaining in feeders and storage bags.

Feed conversion ratio (FCR) was calculated at pen level both on a weekly and cumulative basis as the ratio of cumulative feed intake (g/pen) to cumulative body weight gain (g/pen). Cumulative FCR values were computed for all periods up to day 34 [24].

Water consumption was monitored at pen level throughout the five-day treatment period in Groups I, II, and III to assess whether administration of the EO-based formulation affected voluntary water intake.

### 2.8. Statistical Analysis

The results collected in the study were statistically analysed using Tibco Statistica v13.5 and R software (v4.5.3). Descriptive statistics were used for evaluation of data distribution. Differences between studied experimental groups were evaluated by mixed repeated-measures ANOVA, MANOVA, one-way ANOVA and application of suitable post hoc tests. Similarly, in case of categorical variables Chi square test was used with post hoc calculation of adjusted residuals, while daily faecal scoring data, recorded as a binary outcome (0/1), were analysed using a generalized linear mixed-effects model (GLMM) with a binomial distribution and logit link function, with treatment group and day included as fixed effects and pen included as a random effect to account for repeated observations within the same experimental unit over time. Pairwise comparisons between treatment groups were adjusted using the Holm method. The level of statistical significance was set at *p* = 0.05.

## 3. Results

### 3.1. Chemical Composition of Essential Oils and Physicochemical Properties of Essential Oil-Based Formulation

The detailed chemical profiles of the tested EOs are listed in Supplementary Table S3. A total of 11 compounds, representing 99.92% of oregano EO, and 21 compounds, representing 99.87% of thyme EO, were identified. Oregano EO was predominantly characterized by carvacrol (86.70%), whereas thyme EO contained thymol (35.87%), *p*-cymene (29.98%), and γ-terpinene (17.78%) as its major constituents. The chemical profiles of other tested EOs shown that 18 compounds representing 99.73% of rosemary EO, 25 compounds representing 99.20% of lavender EO, 14 compounds representing 99.96% of cinnamon EO, and 20 compounds representing 99.89% of lemon EO were identified. The rosemary and lavender EOs were dominated by eucalyptol (43.26%) and linalyl acetate (43.09%), respectively. Trans-cinnamaldehyde (79.52%) was the predominant constituent of cinnamon EO, while D-limonene (63.89%) was the major constituent of lemon EO. Besides, quantitative analysis of the developed EO-based formulation showed that it contained 10.25 ± 1.02 g/kg of thymol and 21.66 ± 1.95 g/kg of carvacrol (Table 2).

Droplets size analysis in the obtained EO-based emulsion indicated no statistically significant differences (t(197) = −1.57, *p* = 0.117) between values obtained immediately after emulsion preparation (d = 358.5 ± 51.2 nm; PDI = 0.0204) and values recorded after seven days of storage at 25 °C (d = 370.4 ± 55.6 nm; PDI = 0.0225), thus indicating stability of formulation.

### 3.2. In Vitro Antimicrobial Activity of Essential Oils and Essential Oil-Based Formulation Against Avian Pathogenic E. coli

The MIC values of the six individually tested EOs against the seven MDR-APEC isolates are presented in Table 3.

Oregano EO showed the strongest and most consistent antibacterial activity, with an MIC of 0.003% against all tested isolates, while thyme EO also showed strong inhibitory activity, with MIC values ranging from 0.003% to 0.007%. In contrast, cinnamon, rosemary, lavender, and lemon EOs did not completely inhibit the growth of the tested isolates at concentrations of up to 1%.

Based on the antibacterial activity of the individually tested EOs, oregano and thyme EOs were selected for the preparation of two different EO-based formulations. The MIC values of these two formulations are presented in Table 4.

Both oregano–thyme EO combinations inhibited the growth of all seven MDR-APEC isolates. For all tested isolates, the inhibitory endpoint of Combination 1 was observed at the sixth two-fold dilution of the initial test solution, whereas Combination 2 remained inhibitory up to the seventh two-fold dilution. As Combination 2 retained antibacterial activity at one additional two-fold dilution, it was selected for the development of the EO-based formulation subsequently tested in vivo.

### 3.3. Phenotypic Antimicrobial Resistance Profiles and Virulence Potential of E. coli Isolates During Experimental APEC Infection

The results presented in Table 5 include the antimicrobial resistance profiles and the presence of virulence-associated genes in *E. coli* isolates obtained before and during the experimental APEC infection. Isolates obtained before infection and on days 3, 5, and 19 post-infection differed in terms of the number of antibiotics to which they exhibited resistance, as well as the number of detected virulence-associated genes. The greatest number of different antimicrobial resistance profiles was observed among isolates obtained on day 19 post-infection, whereas the highest number of virulence-associated genes per isolate was recorded among isolates obtained on days 3 and 5 post-infection.

The temporal distributions of resistance to individual antimicrobials and APEC-associated virulence genes are summarized in Supplementary Tables S4 and S5, respectively.

### 3.4. Faecal Consistency Following APEC Challenge

**Figure 1:**
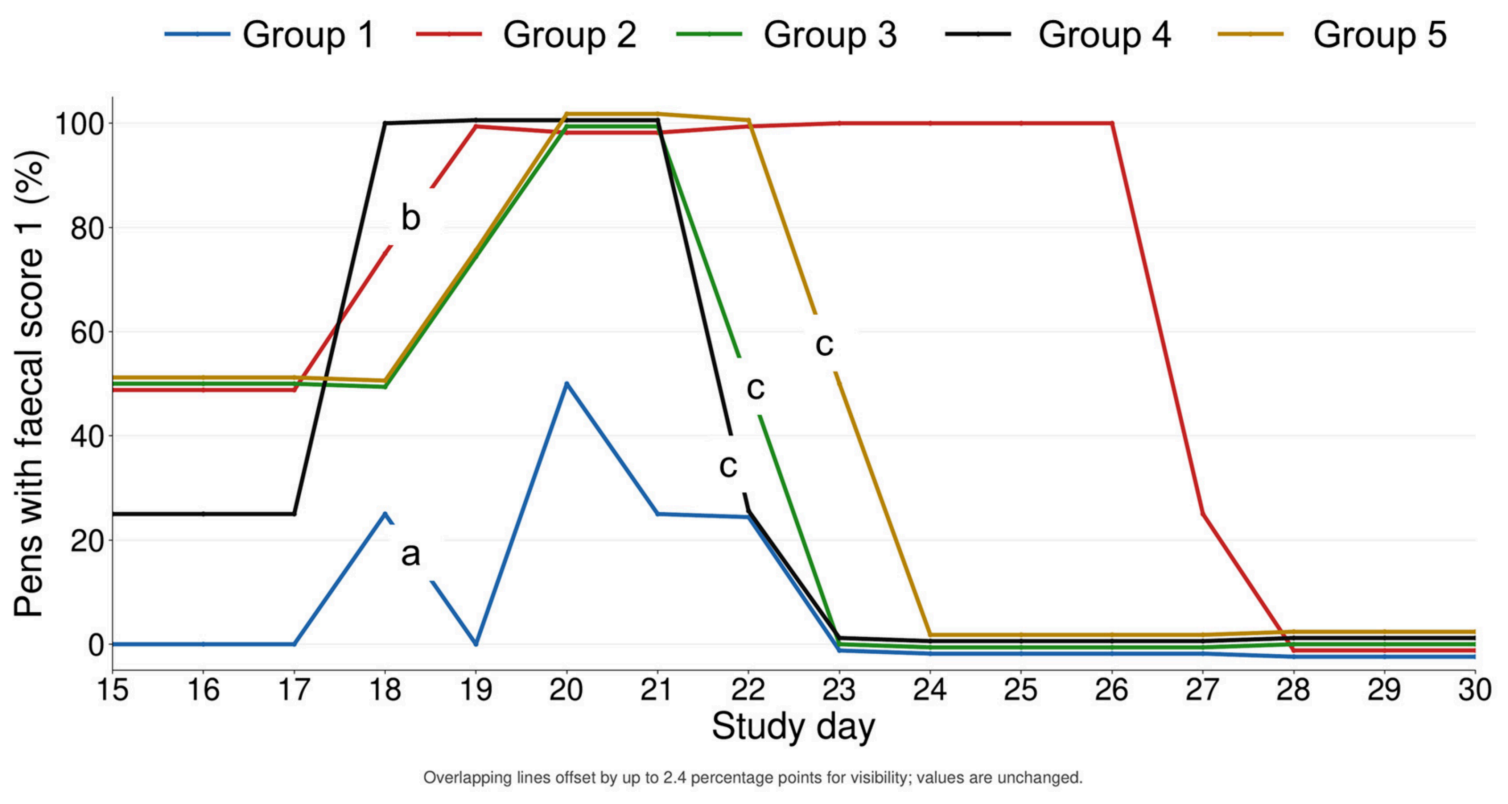
Percentage of pens with a faecal score of 1 across treatment groups from study day 15 to day 30. Groups were assigned different letters based on pairwise comparisons over the entire observation period, whereas groups denoted with different letters differed significantly (binomial GLMM, Holm-adjusted *p* < 0.05).

Daily faecal scoring results (Supplementary Table S6) obtained in the period after infection (from the 15th day of the study period) were analysed using the generalized linear mixed-effects model described above. Statistically significant differences in faecal score were detected between experimental groups (likelihood-ratio χ^2^ = 32.90, df = 4, *p* < 0.001), after accounting for repeated observations within pens, while a significant effect of day was also observed (likelihood-ratio χ^2^ = 177.22, df = 15, *p* < 0.001) (Figure 1).

Pairwise comparisons showed that the negative control group (Group I) differed significantly from the untreated infected group (Group II) and from each treated group (Groups III–V). The untreated infected group (Group II) also differed significantly from each treated group (Groups III–V), whereas no statistically significant differences were found among the EO-treated, antibiotic-treated, and enrofloxacin + reduced-dose EO formulation groups (Table 6).

### 3.5. Growth Performance, Feed Efficiency, and Water Consumption

**Figure 2:**
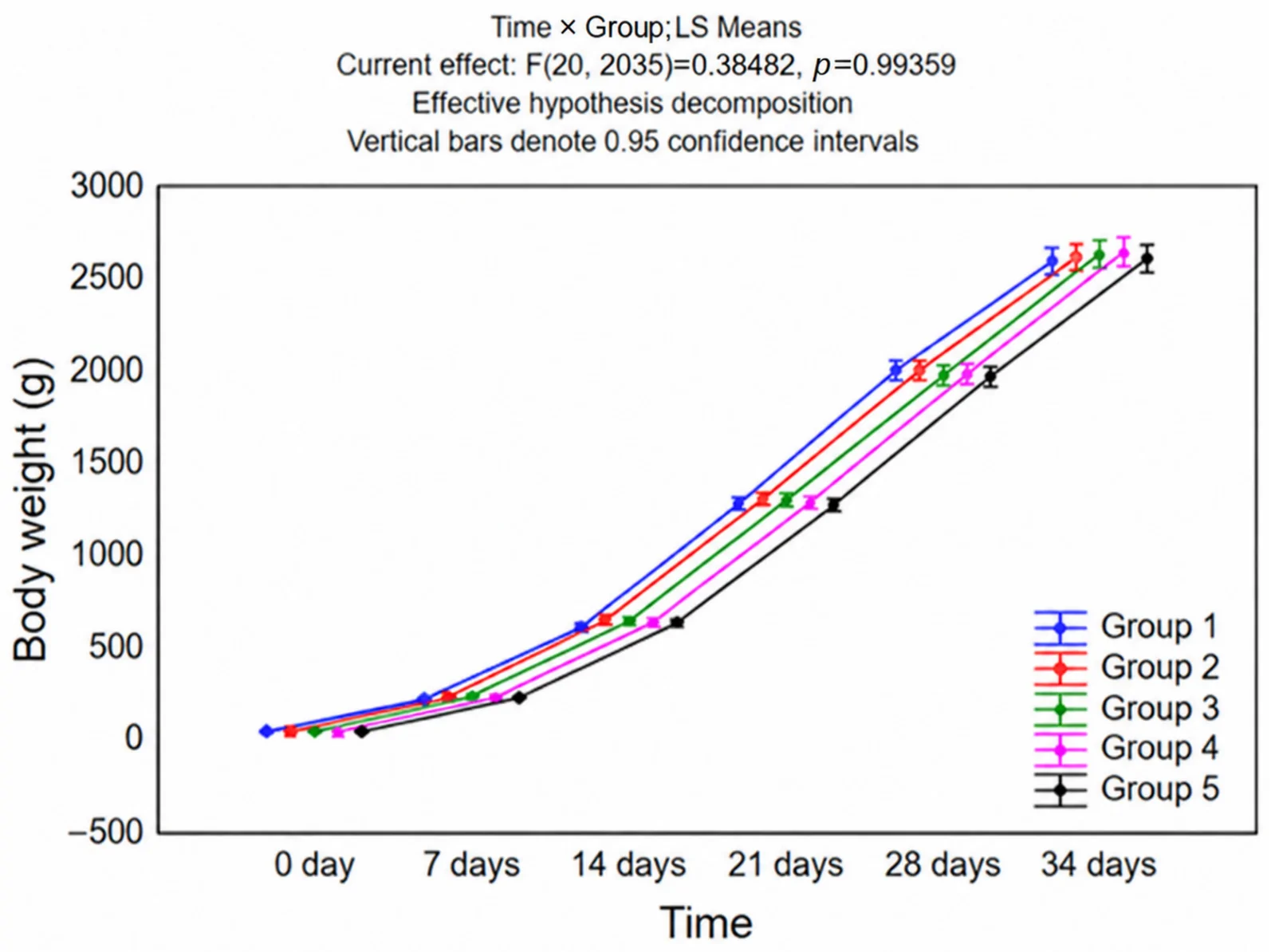
Changes in body weight among experimental groups of broilers during the study period.

The results showed that body weight increased progressively in all experimental groups during the 34-day study period (Figure 2). The application of mixed repeated-measures ANOVA showed that there was no statistically significant difference (F(20, 2035) = 0.38, *p* = 0.99) between experimental groups of broilers regarding pattern of change in body weight during study period. Thus, the pattern of body weight gain over time was comparable among the experimental groups. At the end of the trial, mean body weight ranged from 2588.7 g in the negative control group to 2639.8 g in the antibiotic-treated group (Supplementary Table S7).

**Figure 3:**
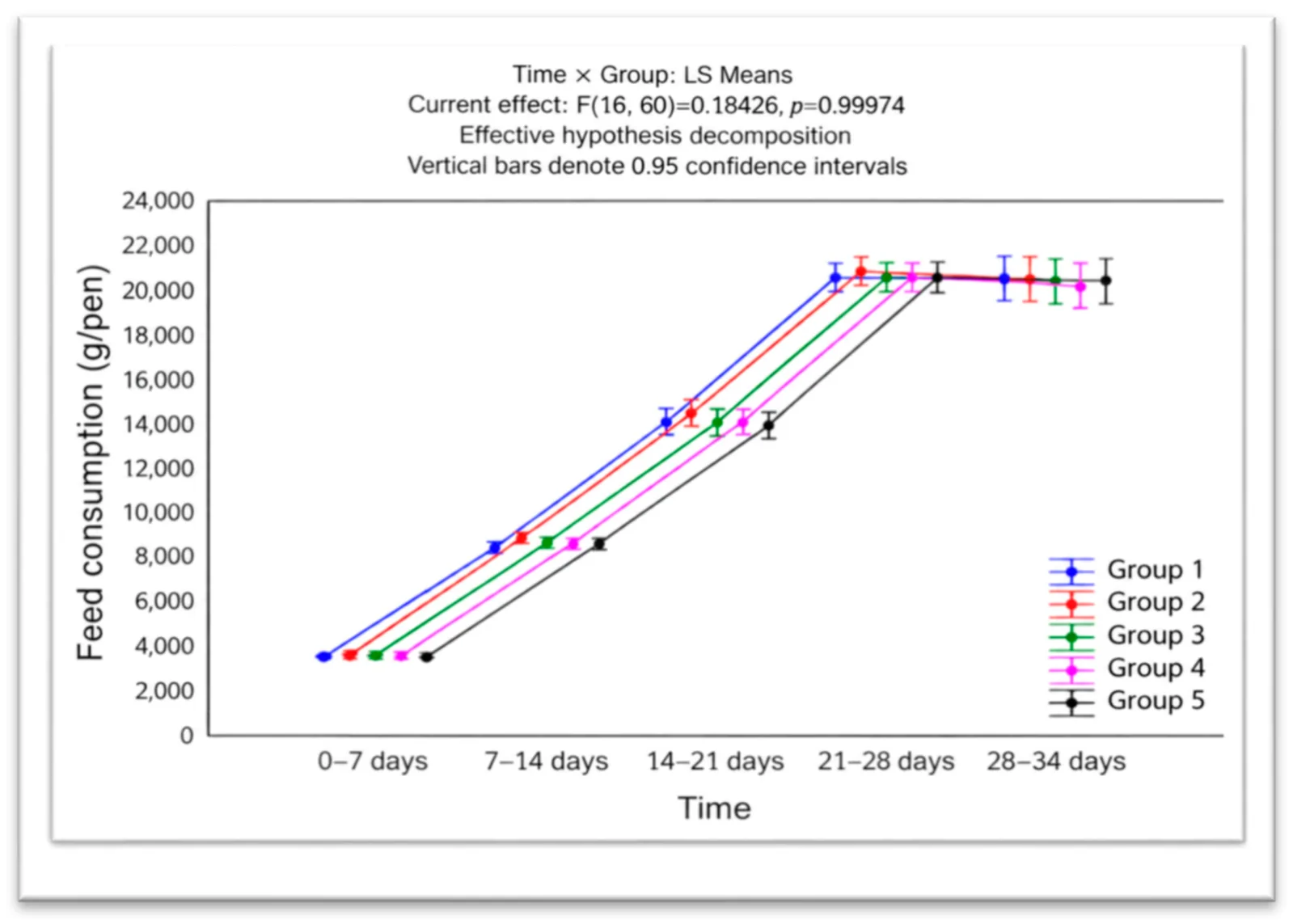
Changes in feed consumption among experimental groups of broilers during the study period.

Feed intake showed a similar pattern in all experimental groups during the study period (Figure 3). The application of mixed repeated-measures ANOVA showed that there was no statistically significant difference (F(16, 60) = 0.18, *p* = 0.99) between experimental groups of broilers regarding pattern of feed consumption during evaluated time intervals. Descriptive data for feed intake across groups and measurement periods are provided in Supplementary Table S8.

**Figure 4:**
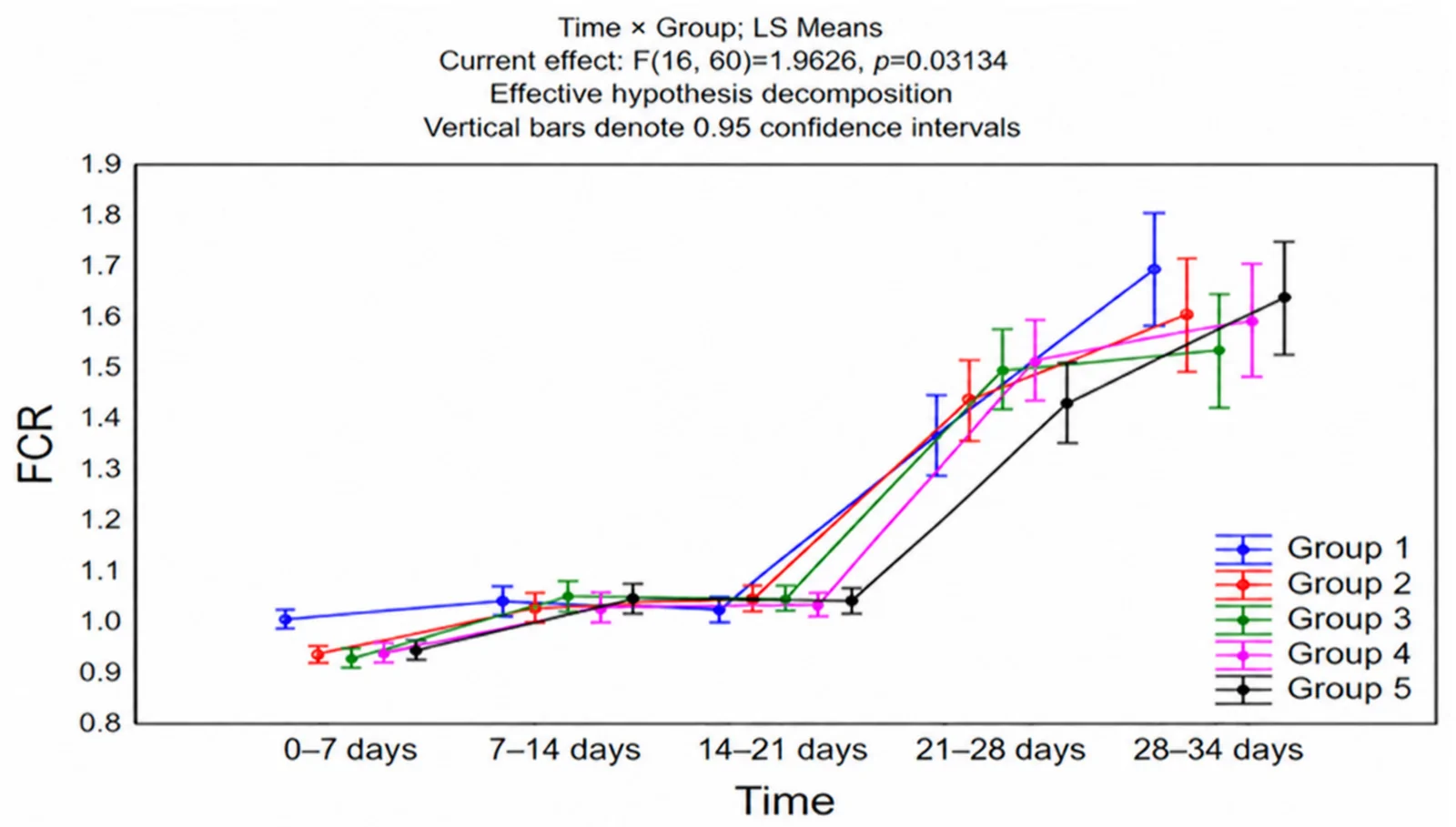
Cumulative changes in feed conversion ratio among experimental groups of broilers during the study period.

Mixed repeated-measures ANOVA identified a significant group-by-time interaction for FCR (F(16, 60) = 1.96, *p* = 0.03), indicating that the temporal pattern of FCR differed among the experimental groups (Figure 4). However, no significant overall treatment effect was observed when FCR values were averaged across the evaluated periods (F = 0.50, *p* = 0.74). Therefore, despite differences in the temporal pattern of FCR, no consistent improvement in feed efficiency attributable to treatment was observed.

**Figure 5:**
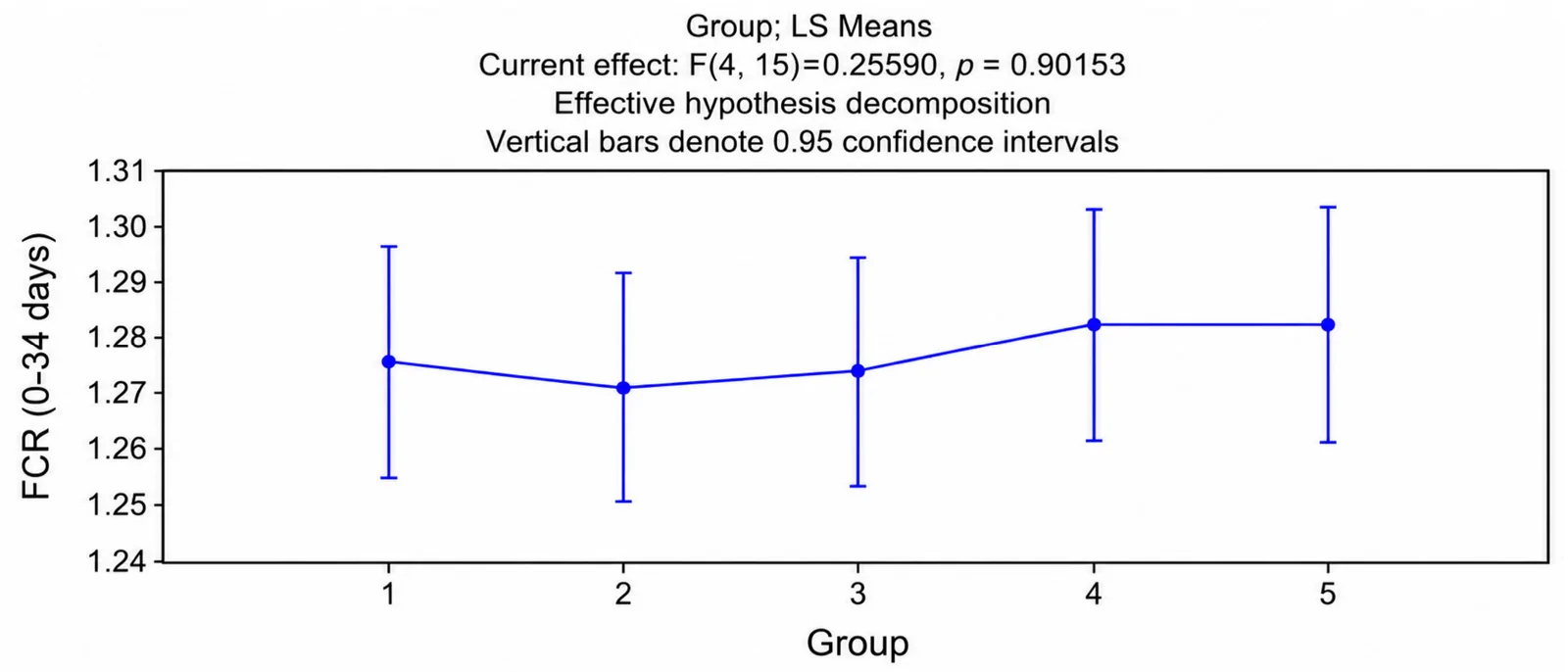
Changes in feed conversion ratio among experimental groups of broilers during the 34-day study period.

Total FCR over the entire 0–34-day study period did not differ significantly among groups (F(4, 15) = 0.26, *p* = 0.90) (Figure 5).

Overall mortality was low, ranging from 0 to 3.57% across the experimental groups, with no significant differences among groups. Mortality rates were 1.19% (1/84), 0% (0/84), 2.38% (2/84), 2.38% (2/84), and 3.57% (3/84) in the negative control, positive control, EO-treated, antibiotic-treated, and enrofloxacin + reduced-dose EO formulation group, respectively.

During the five-day treatment period, mean daily water consumption per pen was 5820 ± 364 mL in Group I, 6241 ± 161 mL in Group II, and 6056 ± 336 mL in the EO-treated Group III. Water intake in the EO-treated group remained within the range observed in the two control groups during the monitored treatment period.

### 3.6. Serum Biochemical Profile

**Figure 6:**
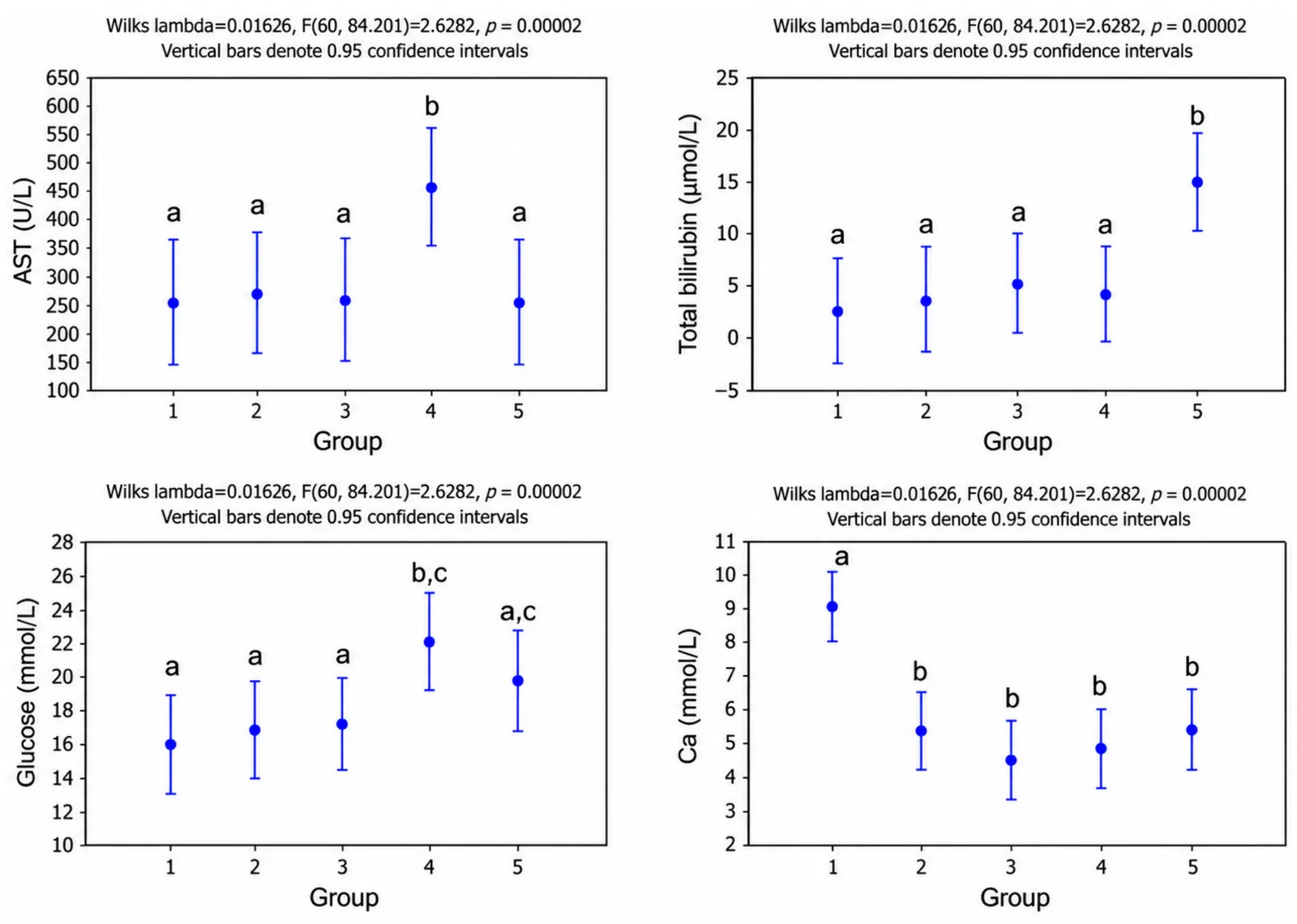
Selected serum biochemical parameters in experimental groups of broilers. Different lowercase letters indicate statistically significant differences between groups (*p* < 0.05); groups sharing at least one letter are not significantly different.

The application of MANOVA on the results of blood biochemical analyses showed a statistically significant difference between studied groups of broilers (Wilk’s λ = 0.002, F(15) = 799.72, *p* = 0.00) when each experimental animal was characterized by complete set of determined biochemical parameters. Follow-up univariate ANOVAs suggested that the difference between groups were significant for AST activity, and total bilirubin, glucose and calcium concentrations (Figure 6). Descriptive statistics for all analysed serum biochemical parameters are provided in Supplementary Table S10.

## 4. Discussion

There is a growing trend in veterinary medicine toward the development of plant-derived pharmaceutical formulations as alternatives to conventional antimicrobial therapy across different animal species and infectious diseases [25,26]. Among these formulations, EO-based formulations have received particular attention, especially because of their potential use against antimicrobial-resistant pathogens [27,28,29,30]. Moreover, previously identified gaps in veterinary practitioners’ knowledge of antimicrobial use and resistance highlight the need to strengthen antimicrobial stewardship and develop effective non-antibiotic interventions [31,32]. However, translating the promising in vitro antimicrobial activity of EOs results into reproducible in vivo efficacy of EO-based formulation results remains challenging, particularly in poultry production. Hence, to address this gap, the present study progressed from in vitro screening of individual EOs and oregano–thyme EO combinations against field-isolated APEC to the development of EO-based pharmaceutical formulation that was further in vivo tested for the controlling of the experimentally induced colibacillosis in broilers.

Chemical characterization of the EOs used in the present study showed that carvacrol and thymol were the predominant secondary metabolites of oregano and thyme EOs, respectively. The predominance of carvacrol in *Origanum vulgare* EO is consist with our previous findings and other published studies reporting that *Origanum vulgare* EO is rich in carvacrol [11,33,34]. Similarly, the chemical profile of *Thymus vulgaris* EO showed thymol as its predominant component in agreement with other studies [11,29]. Furthermore, chemical characterization of the developed EO-based formulation confirmed carvacrol and thymol as predominant secondary metabolites, finding concentrations of 21.66 ± 1.95 g/kg for carvacrol and 10.25 ± 1.02 g/kg for thymol. The predominance of these phenolic monoterpenes provides a promising chemical basis for the antibacterial activity observed against the tested APEC isolates.

Among the six individually tested EOs, oregano and thyme EOs showed the strongest antibacterial activity against the MDR-APEC isolates, with MIC values ranging from 0.003% oregano and 0.003–0.007% thyme, respectively. In contrast, rosemary, cinnamon, lavender, and lemon EOs showed lower inhibitory activity. Based on these findings, oregano and thyme EOs were selected for the preparation of two combinations with the different relative proportion of the two the most antibacterial potent EOs. Both combinations showed antibacterial activity against the tested MDR-APEC isolates, whereas Combination 2 was stronger and more consistent in this activity. This combination is selected for the preparation of EO-based formulation that was further in vivo tested. The antibacterial activity of oregano and thyme EOs and their combinations may be associated with their high contents of carvacrol and thymol. These phenolic monoterpenes disrupt bacterial membranes, increase membrane permeability, and promote the leakage of intracellular components, including ATP and K^+^ ions [9,21]. This multitarget membrane activity may explain why the formulations retained inhibitory effects against APEC isolates, displaying resistance to multiple conventional antimicrobial classes. This mechanism of action may be related to the susceptibility of the APEC isolates to the selected EO combination despite their resistance to multiple conventional antimicrobial classes.

There is a growing trend in the development of pharmaceutical formulations of natural origin, especially those based on EOs, as alternatives to antimicrobials. However, translating in vitro results to in vivo efficacy remains challenging. Hence, this study introduced a phased methodology by first characterizing and screening selected essential oils in vitro against field-isolated APEC. Subsequently, it advanced to formulate the product for drinking water delivery and evaluating its in vivo effects in experimentally infected broilers. Actually, this study aims to bridge that gap by developing a stable, water-administered formulation.

Following promising in vitro results, the selected EO-based formulation was further evaluated in vivo in broilers. The clinical and productive consequences of experimental APEC infection may depend substantially on the inoculation route and the severity of the resulting disease. Kariyawasam et al. (2004) [35] and Horn et al. (2012) [36] reported that intratracheal inoculation with pathogenic *E. coli* strains may facilitate bacterial dissemination from the respiratory tract to other organs, resulting in severe systemic inflammation and impaired production performance. In contrast, the oral challenge applied in the present study produced detectable changes in faecal consistency but did not markedly impair broiler growth performance. The five-day interval between experimental challenge on day 15 and treatment initiation on day 20 was intentionally included to allow the infection to become established and to permit detectable clinical manifestations to develop before therapeutic intervention. By day 20, altered faecal consistency represented the first detectable clinical manifestation of the experimental challenge and provided the practical indication for treatment initiation. Nevertheless, the oral challenge produced a relatively mild clinical course, without pronounced effects on body weight, feed intake, overall FCR, or mortality. Therefore, the present model should not be interpreted as reproducing a severe acute form of colibacillosis, and the in vivo findings should primarily be considered in the context of post-challenge intestinal and clinical responses rather than as evidence of robust acute antibacterial efficacy. Although a significant group-by-time interaction was observed for FCR, this reflected differences in the temporal pattern rather than a consistent overall treatment effect. Overall, none of the applied treatments produced a consistent improvement in body weight, feed intake, or overall FCR. Similar findings have been reported by other authors, who demonstrated that EOs supplementation in broilers either failed to improve production performance or even exerted negative effects on these parameters [37,38,39]. In contrast, several studies have shown that EOs may improve production performance by increasing average body weight and/or improving FCR in broilers experimentally infected with APEC [13,40,41]. These inconsistencies among published studies may be attributed to differences in the type of EOs used, their chemical composition and dosage, as well as variations in the severity of clinical disease and other environmental and management-related factors.

The GLMM analysis of faecal scores revealed significant differences among the experimental groups during the post-challenge period. Direct pairwise comparisons showed that the untreated APEC-challenged group (Group II) differed significantly from each treated group (Groups III–V), with higher odds of a faecal score of 1, whereas no significant differences were observed among the three treated groups. These direct comparisons support an association between treatment and a more favourable faecal score pattern relative to the untreated infected group. Previous studies have demonstrated that APEC infection can induce intestinal mucosal damage, local and systemic inflammation, and alterations in the intestinal microbiota of broiler chickens, supporting the observed changes in faecal consistency in the present study [13,42]. This interpretation is partially supported by previous studies showing that dietary supplementation with bioactive compounds can alleviate intestinal disturbances associated with APEC infection. In addition, Pham et al. (2023) [13] reported that supplementation with a coated essential oil and organic acid mixture improved intestinal health in broilers challenged with *E. coli* O78, including reduced intestinal bacterial load and improved intestinal mucosal characteristics.

An important limitation of the present study is the absence of quantitative assessment of APEC shedding and bacterial loads in target tissues. Although *E. coli* isolates were recovered and characterized during the trial, quantitative culture or qPCR of faecal samples and target organs was not performed. Therefore, the observed differences in faecal consistency should not be interpreted as evidence of bacterial clearance and may also reflect changes in intestinal function or other responses following APEC challenge. Future studies should incorporate quantitative assessment of faecal shedding and bacterial loads in relevant tissues to determine whether these effects are associated with a reduction in bacterial burden and to further characterize the in vivo antibacterial potential of the EO-based formulation.

The recovery of *E. coli* from the negative control group following the experimental challenge was an unexpected finding and represents a potential limitation of the present study. Since Group I was not intentionally exposed to the APEC challenge strain, the detection of isolates carrying multiple virulence-associated genes suggests that unintended exposure may have occurred during the experimental period. Cross-contamination between groups during animal handling, faecal sample collection, or other experimental procedures represents a possible explanation. However, in the absence of molecular confirmation of strain identity, such as whole-genome sequencing, it cannot be conclusively established that the isolates recovered from Group I originated from the challenge strain. The characteristic antimicrobial resistance profile of the challenge strain was not detected among isolates recovered from the negative control group. Therefore, these findings should be interpreted cautiously as potentially representing background intestinal *E. coli* carrying APEC-associated virulence genes or unintended exposure, rather than confirmed transmission of the challenge strain to Group I.

An additional limitation concerns the treatment design of Group V. While Group III received the EO-based formulation at 1 mL/L throughout the treatment period, Group V received the full therapeutic dose of enrofloxacin followed by the EO-based formulation at a reduced concentration of 0.4 mL/L. Consequently, Group V does not represent a direct evaluation of both treatments at their respective full doses, and the present data do not allow conclusions regarding synergistic interactions between enrofloxacin and the EO-based formulation. Future studies specifically designed to assess combined effects should apply equivalent EO exposure across the relevant treatment groups and incorporate an experimental design suitable for evaluating treatment interactions.

Among the biochemical parameters evaluated, significant differences were observed for AST, total bilirubin, glucose, and calcium, whereas the remaining parameters did not differ significantly among the experimental groups. The pattern of these changes suggests that they were not uniformly attributable to APEC infection itself. Serum calcium concentrations were lower in all APEC-challenged groups compared with the non-infected controls, which is consistent with previous studies reporting decreased serum or plasma calcium concentrations following experimental *E. coli* infection in broiler chickens [43,44]. These findings may reflect alterations in calcium homeostasis associated with infection. In contrast, the changes observed in AST, glucose, and total bilirubin appeared to be more closely associated with specific treatment groups. The increase in AST and glucose was observed in broilers treated with enrofloxacin alone, whereas total bilirubin was elevated in birds receiving the enrofloxacin + reduced-dose EO formulation regimen. For additional context, recently established reference intervals for 35-day-old male Ross 308 broilers are 1.4–5.7 µkat/L (approximately 84–342 U/L) for AST, 1.8–7.3 µmol/L for total bilirubin, and 5.8–15.1 mmol/L for glucose [45]. In the present study, mean AST concentrations remained within the reported reference interval in groups I, II, III, and V, whereas the enrofloxacin-treated group (IV) exceeded the upper reference limit, with a mean value of 455.1 U/L. Mean total bilirubin concentrations were within the reported reference interval in groups I–IV, while the enrofloxacin + reduced-dose EO formulation group (V) showed a higher mean value of 14.86 µmol/L. Notably, mean glucose concentrations were above the reported reference interval in all experimental groups, ranging from 15.97 to 22.30 mmol/L, with the highest value observed in the enrofloxacin-treated group. These comparisons should be interpreted with caution because the published reference intervals were established in a specific population of healthy 35-day-old male Ross 308 broilers, whereas the present study included both sexes and serum samples were collected on day 34. Importantly, these changes were not accompanied by consistent alterations in other biochemical indicators of hepatic function, suggesting that they may represent treatment-associated metabolic responses rather than unequivocal evidence of clinically relevant organ dysfunction.

A specific in vitro cytotoxicity assessment and a formal in vivo dose-escalation toxicity study of different EO concentrations were not performed. Therefore, the absence of adverse clinical, productive, or consistent biochemical effects in the EO-treated birds should be interpreted as evidence of tolerability under the applied experimental conditions rather than as a comprehensive toxicological safety evaluation.

## 5. Conclusions

This study translated in vitro screening of six EOs against field-derived MDR-APEC isolates into the development and in vivo evaluation of an oregano–thyme EO-based formulation administered through drinking water. The formulation demonstrated strong antibacterial activity in vitro and was associated with a more favourable faecal score pattern following APEC challenge, while no consistent improvement in productive performance was observed. Because quantitative bacterial shedding or tissue burden was not assessed, these in vivo findings should not be interpreted as evidence of pathogen clearance. The findings support further evaluation of EO-based formulations as potential non-antibiotic approaches for managing APEC-associated intestinal disturbances. Moreover, field trials and pharmacoeconomic analyses are needed to determine their cost-effectiveness and practical value within poultry antimicrobial stewardship programmes.

## Figures and Tables

**Table 1:** Experimental groups, infection status, treatments and treatment period.

Group	*n* (Birds)	Treatment	Infected	Treatment Period (Days)
I—Negative control	84	No treatment	No	–
II—Positive control	84	No treatment	Yes	–
III—EOs	84	EOs formulation (drinking water)	Yes	20–24
IV—Antibiotic	84	Antibiotic (drinking water)	Yes	20–24
V—Enrofloxacin + reduced-dose EO formulation	84	Enrofloxacin + reduced-dose EO formulation (drinking water)	Yes	20–24

**Table 2:** Concentrations of thymol and carvacrol in the developed EO-based formulation. Values are expressed as mean ± standard deviation and reported as grams of compound per kilogram of the final EO-based formulation (g/kg).

Compound	Concentration in the EO-Based Formulation (g/kg)
Thymol	10.25 ± 1.02
Carvacrol	21.66 ± 1.95

**Table 3:** Minimum inhibitory concentrations of individual essential oils against the tested MDR-APEC isolates. Values > 1% indicate that complete inhibition was not observed at the highest concentration tested (1%); the exact MIC above 1% was not determined.

Isolate Number	Oregano	Thyme	Cinnamon	Rosemary	Lavender	Lemon
2416	0.003%	0.003%	>1%	>1%	>1%	>1%
14,857	0.003%	0.007%	>1%	>1%	>1%	>1%
15,912	0.003%	0.007%	>1%	>1%	>1%	>1%
15,913	0.003%	0.007%	>1%	>1%	>1%	>1%
7/2	0.003%	0.007%	>1%	>1%	>1%	>1%
7/10	0.003%	0.007%	>1%	>1%	>1%	>1%
7/24	0.003%	0.003%	>1%	>1%	>1%	>1%

**Table 4:** Inhibitory endpoints of two oregano–thyme EO combinations against the tested MDR-APEC isolates.

Isolate Number	Oregano–Thyme 1	Oregano–Thyme 2
2416	6th two-fold dilution	7th two-fold dilution
14,857	6th two-fold dilution	7th two-fold dilution
15,912	6th two-fold dilution	7th two-fold dilution
15,913	6th two-fold dilution	7th two-fold dilution
7/2	6th two-fold dilution	7th two-fold dilution
7/10	6th two-fold dilution	7th two-fold dilution
7/24	6th two-fold dilution	7th two-fold dilution

Combination 1: oregano–thyme 30:70 (*v*/*v*); Combination 2: oregano–thyme 46.15:53.85 (*v*/*v*).

**Table 5:** Antimicrobial resistance profiles and virulence genes of *E. coli* isolates recovered from pooled faecal samples of the five experimental groups before and after APEC infection.

Sampled	Isolate ID	Resistotype	Virulence Genes
Day 0 before infection	5780/1	AMP	*iutA*
	5780/2	AMP, TET	*iutA*
	5780/3	AMP	*iss*
	5780/4	TET	*iutA*
	5780/5	AMP, TET	*iutA*
Day 3 after infection	6290/1	AMP, TET	*iroN*, *hlyF*, *iutA*, *iss*, *ompT*, *eitC*
	6290/2	AMP, TET	*iroN*, *hlyF*, *iutA*, *iss*, *ompT*, *eitC*
	6290/3	AMP, CIP, NAL, TET, SXT	*iroN*, *iutA*, *iss*, *ompT*, *eitC*
	6290/4	AMP, CIP, NAL	*iroN*, *hlyF*, *iutA*, *iss*, *ompT*, *eitC*
	6290/5	AMP, TET	*iroN*, *hlyF*, *iutA*, *iss*, *ompT*, *cvaC*
Day 5 after infection	6517/1	AMP, TET	*iss*, *ompT*
	6517/2	AMP, TET	*iss*, *ompT*
	6517/3	AMP, CIP, NAL, TET, SXT	*iroN*, *iutA*, *iss*, *ompT*, *eitC*
	6517/4	AMP, CIP, NAL, TET, SXT	*iroN*, *hlyF*, *iutA*, *iss*, *ompT*, *eitC*
	6517/5	AMP, CIP, NAL, TET, SXT	*iroN*, *hlyF*, *iutA*, *iss*, *ompT*, *eitC*
Day 19 after infection	7940/1	TET	*iss*
	7940/2	AMP, CIP, NAL, CHL, STR, TET, SXT	*eitC*
	7940/3	AMP, CIP, NAL, CHL, STR, TET, SXT	*iutA*
	7940/4	AMP, CIP, NAL, CHL, STR, TET, SXT	*iutA*, *cvaC*
	7940/5	AMP, CIP, NAL, STR, TET, SXT	*iroN*, *hlyF*, *iutA*, *iss*, *ompT*, *eitC*

Faecal samples were collected as pooled samples from each experimental group. Isolate IDs ending in /1, /2, /3, /4, and /5 correspond to Groups 1, 2, 3, 4, and 5, respectively. AMP—ampicillin; CIP—ciprofloxacin; NAL—nalidixic acid; TET—tetracycline; STR—streptomycin; CHL—chloramphenicol; SXT—trimethoprim/sulfamethoxazole.

**Table 6:** Pairwise comparisons of treatment groups for binary faecal scores using a binomial generalized linear mixed model.

Comparison Between Groups	Odds Ratio (OR)	95% Confidence Interval for OR	Holm-Adjusted *p*-Value
1 vs. 2	0.0015	0.0002–0.0126	<0.0001
1 vs. 3	0.0307	0.0046–0.2058	0.0012
1 vs. 4	0.0357	0.0054–0.2377	0.0014
1 vs. 5	0.0172	0.0025–0.1191	0.0002
2 vs. 3	20.5667	4.8012–88.1009	0.0014
2 vs. 4	23.8638	5.5013–103.5173	0.0012
2 vs. 5	11.4901	2.8325–46.6097	0.0069
3 vs. 4	1.1603	0.3157–4.2642	0.8230
3 vs. 5	0.5587	0.1548–2.0167	0.8211
4 vs. 5	0.4815	0.1322–1.7531	0.8211

The 95% confidence intervals are unadjusted Wald intervals; *p*-values are based on likelihood-ratio tests and adjusted for 10 comparisons using the Holm method.

## Data Availability

The data presented in this study are available within the article and its Supplementary Materials.

## Supplementary Materials

The following supporting information can be downloaded at: https://www.mdpi.com/article/10.3390/ani16193099/s1, Table S1: Resistotype and virulence genes of the APEC isolates used in the study; Table S2: Primers, annealing temperatures, size of the PCR amplicons and references used in the study [17,18]; Table S3: Results of chemical analysis of evaluated essential oils (n.d.- not detected.); Table S4: Frequency of resistance to individual antimicrobials among *E. coli* isolates recovered before and after experimental APEC infection; Table S5: Distribution of *E. coli* isolates according to the number of virulence genes detected before and after experimental APEC infection; Table S6: Distribution of faecal scores among experimental groups of broilers following APEC challenge; Table S7: Descriptive statistics for body weight of broilers across experimental groups and measurment days; Table S8: Descriptive statistics for feed intake across experimental groups and measurement periods; Table S9: Weekly and cumulative feed conversion ratio across experimental groups of broilers; Table S10: Descriptive statistics for serum biochemical parameters in the experimental groups of broilers.
