# Supplementary material for: From In Vitro Screening to In Vivo Evaluation of Essential-Oil-Based Formulation Against Avian Pathogenic Escherichia coli
Source: Animals (Basel). 2026 Oct 2;16(19):3099. doi: 10.3390/ani16193099 (PMC13663412; doi:10.3390/ani16193099)
Supplement: Supplementary file 1 [file animals-16-03099-s001.zip › S1.Resistotype and virulence genes of the APEC isolates used in the study .pdf]

Supplementary Table S1. Resistotype and virulence genes of the APEC isolates used in the study.

No	Isolate number	Resistotype	Virulence genes
1.	2416	AMP, CHL, CIP, NAL, TET	*iroN*, *ompT, hlyF*, *iss, iutA*, *eitC, cvaC*
2.	14857	AMP, SA, TET, SXT	*iroN*, *ompT, hlyF*, *iss*
3.	15912	AMP, CIP, NAL, TET, SXT	*ompT, iss, iutA*, *eitC*
4.	15913	AMP, CIP, NAL, GEN, TET, SXT	*iutA, eitC*
5.	7/2	AMP, SA, STR, TET, SXT	*iroN*, *ompT, hlyF*, *iss, iutA, cvaC*
6.	7/10	AMP, CHL, SA, STR, TET, SXT	*iroN*, *ompT, hlyF*, *iss*
7.	7/24	AMP, CHL, SA, STR, TET, SXT	*iroN*, *ompT, hlyF*, *iss*

AMP – ampicillin, CHL – chloramphenicol, CIP – ciprofloxacin, NAL – nalidixic acid, GEN – gentamicin, TET – tetracycline, SA – sulfonamides, STR – streptomycin, SXT – trimethoprim/sulfamethoxazole
