# Supplementary material for: From In Vitro Screening to In Vivo Evaluation of Essential-Oil-Based Formulation Against Avian Pathogenic Escherichia coli
Source: Animals (Basel). 2026 Oct 2;16(19):3099. doi: 10.3390/ani16193099 (PMC13663412; doi:10.3390/ani16193099)
Supplement: Supplementary file 1 [file animals-16-03099-s001.zip › S10. Descriptive statistics for serum biochemical parameters in the experimental groups of broilers..pdf]

Supplementary Table S10. Descriptive statistics for serum biochemical parameters in the experimental groups of broilers.

Variable	Group	N	Mean	Median	Minimum	Maximum	Lower Quartile	Upper Quartile	Percentile 5.0	Percentile 95.0	Variance	Std. Dev.	Confidence SD -95.000%	Confidence SD +95.000%
Albumin (g/L)	I	8	21.9375	23.0000	12.00000	31.2000	18.7000	24.4500	12.00000	31.2000	31.90	5.6482	3.73447	11.4957
ALT (U/L)	I	8	4.6425	2.7250	1.92000	13.9000	2.2800	5.6550	1.92000	13.9000	16.31	4.0389	2.67045	8.2204
AST (U/L	I	8	252.7500	251.5000	54.00000	411.0000	181.5000	345.5000	54.00000	411.0000	14471.93	120.2993	79.53881	244.8419
Total bilirubin (µmol/L)	I	8	2.7000	2.5850	0.70000	5.4000	1.2400	3.9250	0.70000	5.4000	2.99	1.7296	1.14354	3.5201
Cholesterol (mmol/L)	I	8	2.7675	2.7000	1.46000	4.1400	2.2050	3.3650	1.46000	4.1400	0.72	0.8495	0.56166	1.7289
Creatinine (µmol/L)	I	8	49.4738	49.0500	46.30000	56.6200	46.9400	50.4450	46.30000	56.6200	11.12	3.3344	2.20461	6.7864
GGT (U/L)	I	8	15.2525	13.3500	7.27000	27.1000	10.6000	19.8750	7.27000	27.1000	49.55	7.0390	4.65402	14.3263
Glucose (mmol/L)	I	8	15.9688	16.1500	13.50000	18.5000	14.5000	17.2250	13.50000	18.5000	2.90	1.7036	1.12635	3.4672
Ca (mmol/L)	I	8	9.1300	8.7000	6.30000	12.8000	8.5250	9.7450	6.30000	12.8000	3.35	1.8314	1.21085	3.7273
Mg (mmol/L)	I	8	0.8375	0.8150	0.72000	1.0800	0.7750	0.8600	0.72000	1.0800	0.01	0.1087	0.07189	0.2213
P (mmol/L)	I	8	5.1263	5.1550	4.20000	6.0000	4.4500	5.8000	4.20000	6.0000	0.51	0.7136	0.47184	1.4525
Total protein (g/L)	I	8	33.3425	32.7000	23.50000	42.5400	28.8000	38.8500	23.50000	42.5400	46.00	6.7824	4.48436	13.8041
Triglycerides (mmol/L)	I	8	0.9555	0.8900	0.59000	1.4010	0.8125	1.1240	0.59000	1.4010	0.07	0.2569	0.16986	0.5229
Urea (mmol/L)	I	8	0.4914	0.4705	0.18000	0.8020	0.3770	0.6270	0.18000	0.8020	0.04	0.1969	0.13019	0.4008
Globulin (g/L)	I	8	11.2688	9.1950	3.65000	22.1000	5.5200	17.4850	3.65000	22.1000	50.08	7.0767	4.67896	14.4031
Albumin (g/L)	II	8	24.1500	26.7000	11.40000	29.4000	21.3500	28.1500	11.40000	29.4000	36.15	6.0124	3.97522	12.2368
ALT (U/L)	II	8	2.7800	2.8000	1.60000	3.9400	2.0500	3.5000	1.60000	3.9400	0.71	0.8406	0.55579	1.7109
AST (U/L	II	8	270.0000	305.5000	43.00000	367.0000	252.0000	317.5000	43.00000	367.0000	10522.29	102.5782	67.82205	208.7745
Total bilirubin (µmol/L)	II	8	3.5988	2.2600	0.45000	11.0000	1.2350	5.1750	0.45000	11.0000	12.82	3.5801	2.36710	7.2866
Cholesterol (mmol/L)	II	8	2.5075	2.4350	1.81000	3.6300	1.8600	3.0150	1.81000	3.6300	0.47	0.6847	0.45270	1.3935
Creatinine (µmol/L)	II	8	48.1163	48.5050	41.98000	56.4700	43.9300	50.8050	41.98000	56.4700	23.15	4.8110	3.18094	9.7918
GGT (U/L)	II	8	15.4515	15.8750	6.58200	23.1500	12.6300	18.4350	6.58200	23.1500	25.12	5.0117	3.31357	10.2001
Glucose (mmol/L)	II	8	16.7375	16.4500	13.70000	20.2000	15.2000	18.3500	13.70000	20.2000	4.67	2.1620	1.42944	4.4002
Ca (mmol/L)	II	8	5.3688	4.3650	3.13000	10.5000	4.2500	6.0450	3.13000	10.5000	5.77	2.4026	1.58855	4.8900
Mg (mmol/L)	II	8	0.8400	0.8050	0.70000	1.0700	0.7350	0.9350	0.70000	1.0700	0.02	0.1303	0.08613	0.2651
P (mmol/L)	II	8	4.1963	4.1700	2.72000	5.1400	3.7950	4.8900	2.72000	5.1400	0.65	0.8050	0.53223	1.6384
Total protein (g/L)	II	8	32.6500	32.2000	27.20000	39.1000	29.0500	36.2000	27.20000	39.1000	19.17	4.3779	2.89453	8.9101
Triglycerides (mmol/L)	II	8	0.7500	0.6850	0.49000	1.1700	0.5200	0.9650	0.49000	1.1700	0.07	0.2704	0.17881	0.5504
Urea (mmol/L)	II	8	0.4074	0.3830	0.26800	0.5740	0.3325	0.4930	0.26800	0.5740	0.01	0.1042	0.06889	0.2121

Variable	Group	N	Mean	Median	Minimum	Maximum	Lower Quartile	Upper Quartile	Percentile 5.0	Percentile 95.0	Variance	Std. Dev.	Confidence SD -95.000%	Confidence SD +95.000%
Globulin (g/L)	II	8	8.5025	8.2000	-2.20000	18.7200	2.9500	14.6000	-2.20000	18.7200	52.52	7.2472	4.79163	14.7499
Albumin (g/L)	III	8	21.1750	21.2000	18.2000	26.9000	18.8000	22.1500	18.2000	26.9000	7.98	2.8253	1.86799	5.7502
ALT (U/L)	III	8	3.2125	3.2500	1.8000	4.5000	2.3500	4.1000	1.8000	4.5000	1.10	1.0480	0.69294	2.1331
AST (U/L	III	8	256.8750	281.5000	113.0000	375.0000	154.5000	347.5000	113.0000	375.0000	10869.55	104.2572	68.93213	212.1917
Total bilirubin (µmol/L)	III	8	5.2300	4.5000	2.4700	11.7000	3.6100	5.7250	2.4700	11.7000	8.18	2.8602	1.89111	5.8214
Cholesterol (mmol/L)	III	8	2.5788	2.4900	1.9200	3.2800	2.2600	2.9650	1.9200	3.2800	0.21	0.4603	0.30431	0.9368
Creatinine (µmol/L)	III	8	47.5500	47.3850	43.1200	51.4700	45.3400	50.1800	43.1200	51.4700	8.92	2.9860	1.97423	6.0772
GGT (U/L)	III	8	17.5950	17.5650	5.5400	27.3000	12.7750	23.6200	5.5400	27.3000	53.32	7.3022	4.82804	14.8620
Glucose (mmol/L)	III	8	17.3375	15.6000	13.6000	24.9000	14.9000	19.6000	13.6000	24.9000	14.21	3.7690	2.49198	7.6710
Ca (mmol/L)	III	8	4.6375	4.7000	4.1000	4.9900	4.4700	4.8350	4.1000	4.9900	0.08	0.2842	0.18790	0.5784
Mg (mmol/L)	III	8	0.8863	0.9000	0.7800	0.9500	0.8450	0.9350	0.7800	0.9500	0.00	0.0595	0.03934	0.1211
P (mmol/L)	III	8	5.7938	5.4800	2.6100	11.2000	3.3250	7.4650	2.6100	11.2000	9.02	3.0032	1.98561	6.1122
Total protein (g/L)	III	8	34.3750	34.7500	28.6000	41.0000	31.0500	36.9000	28.6000	41.0000	16.82	4.1011	2.71156	8.3469
Triglycerides (mmol/L)	III	8	0.8988	0.8750	0.5400	1.5400	0.6650	1.0150	0.5400	1.5400	0.10	0.3174	0.20984	0.6459
Urea (mmol/L)	III	8	0.3901	0.3870	0.2650	0.5790	0.2765	0.4750	0.2650	0.5790	0.01	0.1205	0.07969	0.2453
Globulin (g/L)	III	8	13.2000	12.2500	8.6000	20.3000	9.6500	16.4500	8.6000	20.3000	19.43	4.4084	2.91474	8.9724
Albumin (g/L)	IV	8	22.2750	21.7500	17.50000	30.0000	20.5500	23.0500	17.50000	30.0000	13.01	3.6062	2.3844	7.3397
ALT (U/L)	IV	8	3.1875	2.7500	2.10000	6.2000	2.3500	3.5000	2.10000	6.2000	1.78	1.3336	0.8817	2.7142
AST (U/L	IV	8	455.1250	490.5000	60.00000	744.0000	287.5000	640.5000	60.00000	744.0000	59762.41	244.4635	161.6330	497.5498
Total bilirubin (µmol/L)	IV	8	4.4325	2.0250	0.20000	16.0000	1.0850	6.5200	0.20000	16.0000	28.78	5.3651	3.5473	10.9195
Cholesterol (mmol/L)	IV	8	2.8150	2.7500	2.20000	4.1000	2.3500	3.0100	2.20000	4.1000	0.38	0.6145	0.4063	1.2508
Creatinine (µmol/L)	IV	8	48.4388	48.1950	38.62000	55.2100	46.8000	51.8450	38.62000	55.2100	26.29	5.1271	3.3899	10.4351
GGT (U/L)	IV	8	17.8813	18.5650	11.32000	22.6000	13.7000	22.3000	11.32000	22.6000	20.69	4.5489	3.0076	9.2582
Glucose (mmol/L)	IV	8	22.3000	21.9500	14.20000	34.8000	17.3000	25.4500	14.20000	34.8000	43.35	6.5844	4.3534	13.4010
Ca (mmol/L)	IV	8	4.7938	4.6750	4.20000	5.6500	4.5200	5.0550	4.20000	5.6500	0.24	0.4879	0.3226	0.9930
Mg (mmol/L)	IV	8	0.7539	0.7100	0.47000	1.0300	0.6570	0.8985	0.47000	1.0300	0.04	0.1909	0.1262	0.3886
P (mmol/L)	IV	8	4.2738	4.1500	2.70000	6.2000	3.4950	5.0000	2.70000	6.2000	1.35	1.1614	0.7679	2.3637
Total protein (g/L)	IV	8	33.3625	32.8500	27.20000	42.3000	27.9000	37.9500	27.20000	42.3000	34.91	5.9086	3.9066	12.0256
Triglycerides (mmol/L)	IV	8	1.1975	1.1350	0.63000	1.9400	0.7600	1.6100	0.63000	1.9400	0.25	0.4981	0.3293	1.0137
Urea (mmol/L)	IV	8	0.3860	0.3300	0.26700	0.7500	0.2780	0.4275	0.26700	0.7500	0.03	0.1612	0.1066	0.3281
Globulin (g/L)	IV	8	11.0875	10.7500	-1.80000	20.9000	7.4500	16.6000	-1.80000	20.9000	54.16	7.3596	4.8660	14.9788
Albumin (g/L)	V	8	20.6250	21.3500	15.00000	23.3000	19.4500	22.5500	15.00000	23.3000	7.29	2.6991	1.78456	5.4933

Variable	Group	N	Mean	Median	Minimum	Maximum	Lower Quartile	Upper Quartile	Percentile 5.0	Percentile 95.0	Variance	Std. Dev.	Confidence SD -95.000%	Confidence SD +95.000%
ALT (U/L)	V	8	4.1125	3.6500	1.90000	7.2000	2.2500	6.0000	1.90000	7.2000	4.16	2.0406	1.34920	4.1532
AST (U/L	V	8	253.6250	261.5000	79.00000	362.0000	181.0000	351.5000	79.00000	362.0000	10786.55	103.8583	68.66845	211.3800
Total bilirubin (µmol/L)	V	8	14.8625	11.5500	1.50000	38.9000	4.8000	22.9000	1.50000	38.9000	166.13	12.8891	8.52193	26.2328
Cholesterol (mmol/L)	V	8	2.4338	2.4400	1.72000	3.2500	2.0600	2.7500	1.72000	3.2500	0.24	0.4927	0.32577	1.0028
Creatinine (µmol/L)	V	8	44.9163	44.5900	38.62000	53.4400	41.6550	47.3900	38.62000	53.4400	22.07	4.6974	3.10578	9.5604
GGT (U/L)	V	8	17.7125	17.7000	14.01000	22.9400	15.5650	19.1100	14.01000	22.9400	7.87	2.8060	1.85529	5.7111
Glucose (mmol/L)	V	8	19.5250	18.6000	15.80000	27.0000	17.8500	20.2500	15.80000	27.0000	11.50	3.3906	2.24180	6.9009
Ca (mmol/L)	V	8	5.4163	5.4000	4.80000	6.2200	5.1700	5.5850	4.80000	6.2200	0.18	0.4287	0.28343	0.8725
Mg (mmol/L)	V	8	0.7784	0.7550	0.55000	1.1200	0.5915	0.9320	0.55000	1.1200	0.04	0.2116	0.13991	0.4307
P (mmol/L)	V	8	4.6525	5.0750	1.87000	7.1100	3.2700	5.7750	1.87000	7.1100	3.00	1.7307	1.14431	3.5225
Total protein (g/L)	V	8	30.2625	29.9000	25.50000	34.7000	27.7000	33.3500	25.50000	34.7000	10.99	3.3144	2.19142	6.7458
Triglycerides (mmol/L)	V	8	0.8538	0.8150	0.47000	1.2200	0.6650	1.0900	0.47000	1.2200	0.07	0.2633	0.17410	0.5359
Urea (mmol/L)	V	8	0.3971	0.4030	0.18300	0.5300	0.3750	0.4540	0.18300	0.5300	0.01	0.1015	0.06713	0.2066
Globulin (g/L)	V	8	9.6375	10.5000	2.90000	14.0000	7.4500	12.1500	2.90000	14.0000	13.98	3.7386	2.47185	7.6090
