# Supplementary material for: From In Vitro Screening to In Vivo Evaluation of Essential-Oil-Based Formulation Against Avian Pathogenic Escherichia coli
Source: Animals (Basel). 2026 Oct 2;16(19):3099. doi: 10.3390/ani16193099 (PMC13663412; doi:10.3390/ani16193099)
Supplement: Supplementary file 1 [file animals-16-03099-s001.zip › S2. Primers, annealing temperature, size of the PCR amplicons and references used in the stud.pdf]

Supplementary Table S2. Primers, annealing temperature, size of the PCR amplicons and references used in the study [17,18].

Target genes	Primer sequences 5'to 3'	Anniling temp.	Fragment sizes	References
iroN	fw: AAT CCG GCA AAG AGA CGA ACC GCC T	63°C	553 bp	Johnson et al. 2008.
	rv: GTT CGG GCA ACC CCT GCT TTG ACT TT			
ompT	fw: TCA TCC CGG AAG CCT CCC TCA CTA CTA T	63°C	496 bp	Johnson et al. 2008.
	rv: TAG CGT TTG CTG CAC TGG CTT CTG ATA C			
hlyF	fw: GGC CAC AGT CGT TTA GGG TGC TTA CC	63°C	450 bp	Johnson et al. 2008.
	rv: GGC GGT TTA GGC ATT CCG ATA CTC AG			
iss	fw: CAG CAA CCC GAA CCA CTT GAT G	63°C	323 bp	Johnson et al. 2008.
	rv: AGC ATT GCC AGA GCG GCA GAA			
iutA	fw: GGC TGG ACA TCA TGG GAA CTG G	63°C	302 bp	Johnson et al. 2008.
	rv: CGT CGG GAA CGG GTA GAA TCG			
eitC	fw: CAG CAG CGC TTC GGA CAA AAT CTC C	63°C	380 bp	Johnson et al. 2010.
	rv: TTC CCC ACC ACT CTC CGT TCT CAA AC			
cvaC	fw: CAC ACA CAA ACG GGA GCT GTT	63°C	678 bp	Johnson et al. 2010.
	rv: CTT CCC GCA GCA TAG TTC CAT			
