# Supplementary material for: From In Vitro Screening to In Vivo Evaluation of Essential-Oil-Based Formulation Against Avian Pathogenic Escherichia coli
Source: Animals (Basel). 2026 Oct 2;16(19):3099. doi: 10.3390/ani16193099 (PMC13663412; doi:10.3390/ani16193099)
Supplement: Supplementary file 1 [file animals-16-03099-s001.zip › S3. The results of chemical analysis of evaluated essential oils .pdf]

Supplementary Table S3. The results of chemical analysis of evaluated essential oils (n.d. – not detected).

Compound (%)	Essential oil					
	Oregano	Thyme	Rosemary	Lavender	Cinnamon	Lemon
Tricyclene	n. d.	n. d.	0.41	n. d.	n. d.	n. d.
α-Thujene	n. d.	n. d.	n. d.	n. d.	n. d.	0.54
D-α-Pinene	0.07	0.99	10.02	0.53	0.04	2.42
Camphene	n. d.	n. d.	4.9	0.34	0.21	0.15
Benzaldehyde	n. d.	n. d.	n. d.	n. d.	1.48	n. d.
β-Phellandrene	n. d.	0.14	n. d.	n. d.	n. d.	1.93
β-Pinene	0.13	0.2	7.3	0.27	0.06	12.84
β-Myrcene	n. d.	0.19	1.11	0.35	n. d.	1.4
δ-2-Carene	0.07	n. d.	n. d.	n. d.	n. d.	n. d.
α-Phellandrene	n. d.	0.12	n. d.	n. d.	n. d.	0.08
3-Carene	1.74	0.12	0.31	n. d.	n. d.	n. d.
Hexyl acetate	n. d.	n. d.	n. d.	0.34	n. d.	n. d.
α-Terpinene	0.17	1.24	n. d.	n. d.	n. d.	0.3
p-Cymene	1.78	29.98	1.65	n. d.	1.14	1.22
D-Limonene	1.41	0.17	n. d.	0.44	0.71	63.89
Eucalyptol	n. d.	0.26	43.26	3.94	n. d.	n. d.
trans-β-Ocimene	n. d.	n. d.	n. d.	0.33	n. d.	n. d.
Benzyl alcohol	n. d.	n. d.	n. d.	n. d.	15.12	n. d.
β-Ocimene	n. d.	n. d.	n. d.	0.6	n. d.	0.11
γ-Terpinene	2.09	17.78	n. d.	n. d.	0.66	9.51
Terpinolene	0.89	0.28	0.4	n. d.	n. d.	0.47
Linalool	n. d.	4.13	1.22	33.17	0.33	n. d.
1-Octen-3-yl-acetate	n. d.	n. d.	n. d.	0.3	n. d.	n. d.
1,2-Dihydrolinalool	n. d.	n. d.	n. d.	0.23	n. d.	n. d.
cis-Limonene oxide	n. d.	n. d.	n. d.	n. d.	n. d.	0.11
Camphor	n. d.	n. d.	11.97	4.26	n. d.	n. d.
Isoborneol	n. d.	n. d.	0.1	1.03	n. d.	n. d.
endo-Borneol	n. d.	n. d.	5.1	2.21	n. d.	n. d.
Terpinen-4-ol	n. d.	n. d.	1.37	0.35	n. d.	n. d.
Hexyl butyrate	n. d.	n. d.	n. d.	0.66	n. d.	n. d.
α-Terpineol	n. d.	0.32	3.08	n. d.	n. d.	n. d.
cis-Cinnamaldehyde	n. d.	n. d.	n. d.	n. d.	0.33	n. d.
β-Citral	n. d.	n. d.	n. d.	n. d.	n. d.	1.19
Linalyl acetate	n. d.	n. d.	n. d.	43.09	n. d.	n. d.
α-Citral	n. d.	n. d.	n. d.	n. d.	n. d.	2.31
trans-Cinnamaldehyde	n. d.	n. d.	n. d.	n. d.	79.52	n. d.
Bornyl acetate	n. d.	n. d.	1.66	n. d.	n. d.	n. d.
Lavandulyl acetate	n. d.	n. d.	n. d.	0.19	n. d.	n. d.
Thymol	4.87	35.87	n. d.	n. d.	n. d.	n. d.
Carvacrol	86.7	3.81	n. d.	n. d.	n. d.	n. d.
α-Cubebene	n. d.	0.12	n. d.	n. d.	n. d.	n. d.

Compound (%)	Essential oil					
	Oregano	Thyme	Rosemary	Lavender	Cinnamon	Lemon
Nerol acetate	n. d.	n. d.	n. d.	0.24	n. d.	0.61
α-Copaene	n. d.	0.26	n. d.	0.12	0.05	n. d.
Neryl propionate	n. d.	n. d.	n. d.	0.38	n. d.	0.51
Caryophyllene	n. d.	3.46	5.79	5.56	0.24	n. d.
trans-α-Bergamotene	n. d.	n. d.	n. d.	n. d.	n. d.	0.13
Humulene	n. d.	0.16	n. d.	n. d.	n. d.	n. d.
cis-β-Farnesene	n. d.	n. d.	n. d.	0.12	n. d.	n. d.
β-Bisabolene	n. d.	n. d.	n. d.	n. d.	n. d.	0.17
Caryophyllene oxide	n. d.	0.27	0.08	0.15	0.07	n. d.
