# Supplementary material for: From In Vitro Screening to In Vivo Evaluation of Essential-Oil-Based Formulation Against Avian Pathogenic Escherichia coli
Source: Animals (Basel). 2026 Oct 2;16(19):3099. doi: 10.3390/ani16193099 (PMC13663412; doi:10.3390/ani16193099)
Supplement: Supplementary file 1 [file animals-16-03099-s001.zip › S4. Frequency of resistance to individual antimicrobials among E. coli isolates recovered before and after.pdf]

**Supplementary Table S4.** Frequency of resistance to individual antimicrobials among *E. coli* isolates recovered before and after experimental APEC infection.

| Sampled | Number of isolates | AMP | TET | CIP | NAL | SXT | CHL | STR |
|---|---|---|---|---|---|---|---|---|
| Day 0 | 5 | 3 | 3 | 0 | 0 | 0 | 0 | 0 |
| Day 3 | 5 | 5 | 4 | 2 | 2 | 1 | 0 | 0 |
| Day 5 | 5 | 5 | 5 | 3 | 3 | 3 | 0 | 0 |
| Day 19 | 5 | 4 | 5 | 4 | 4 | 4 | 3 | 4 |

AMP - ampicillin; CIP - ciprofloxacin; NAL – nalidixic acid; TET - tetracycline; STR - streptomycin; CHL - chloramphenicol; SXT - trimethoprim/sulfamethoxazole
