# Supplementary material for: From In Vitro Screening to In Vivo Evaluation of Essential-Oil-Based Formulation Against Avian Pathogenic Escherichia coli
Source: Animals (Basel). 2026 Oct 2;16(19):3099. doi: 10.3390/ani16193099 (PMC13663412; doi:10.3390/ani16193099)
Supplement: Supplementary file 1 [file animals-16-03099-s001.zip › S5. Distribution of E. coli isolates according to the number of virulence genes detected before and after .pdf]

Supplementary Table S5. Distribution of *E. coli* isolates according to the number of virulence genes detected before and after experimental APEC infection.

APEC infection

Virulence genes	Day 0	Day 3	Day 5	Day 19
iroN	0/5	5/5	3/5	1/5
iutA	3/5	5/5	3/5	3/5
iss	1/5	5/5	5/5	2/5
hlyF	0/5	4/5	2/5	1/5
ompT	0/5	4/5	5/5	1/5
eitC	0/5	4/5	4/5	2/5
cvaC	0/5	1/5	0/5	1/5
