# Supplementary material for: From In Vitro Screening to In Vivo Evaluation of Essential-Oil-Based Formulation Against Avian Pathogenic Escherichia coli
Source: Animals (Basel). 2026 Oct 2;16(19):3099. doi: 10.3390/ani16193099 (PMC13663412; doi:10.3390/ani16193099)
Supplement: Supplementary file 1 [file animals-16-03099-s001.zip › S6. Distribution of faecal scores among experimental groups of broilers following APEC challenge.pdf]

Supplementary Table S6. Distribution of faecal scores among experimental groups of broilers following APEC challenge.

Faecal score	Experimental group	Count	Cumulative count	Percent	Cumulative Percent
0	I	59	59	92.18750	92.1875
1	I	5	64	7.81250	100.0000
0	II	22	22	34.37500	34.3750
1	II	42	64	65.62500	100.0000
0	III	43	43	67.18750	67.1875
1	III	21	64	32.81250	100.0000
0	IV	44	44	68.75000	68.7500
1	IV	20	64	31.25000	100.0000
0	V	39	39	60.93750	60.9375
1	V	25	64	39.06250	100.0000
