# Supplementary material for: From In Vitro Screening to In Vivo Evaluation of Essential-Oil-Based Formulation Against Avian Pathogenic Escherichia coli
Source: Animals (Basel). 2026 Oct 2;16(19):3099. doi: 10.3390/ani16193099 (PMC13663412; doi:10.3390/ani16193099)
Supplement: Supplementary file 1 [file animals-16-03099-s001.zip › S7. Descriptive statistics for body weight of broilers across experimental groups and measur.pdf]

**Supplementary Table S7.** Descriptive statistics for body weight of broilers across experimental groups and measurement days.

| Variable | Group | N | Mean | Median | Minimum | Maximum | Lower Quartile | Upper Quartile | Percentile 5.0 | Percentile 95.0 | Variance | Std. Dev. | Confidence SD -95.000% | Confidence SD +95.000% |
|---|---|---|---|---|---|---|---|---|---|---|---|---|---|---|
| m_0days | I | 84 | 45.826 | 45.435 | 39.020 | 58.450 | 43.310 | 48.230 | 39.930 | 52.450 | 14.6 | 3.8205 | 3.3173 | 4.5051 |
| m_7days | I | 84 | 215.833 | 210.000 | 160.000 | 270.000 | 197.500 | 235.000 | 175.000 | 260.000 | 645.7 | 25.4103 | 22.0635 | 29.9634 |
| m_14days | I | 84 | 606.583 | 604.000 | 330.000 | 760.000 | 562.500 | 667.500 | 485.000 | 730.000 | 6838.8 | 82.6969 | 71.8049 | 97.5149 |
| m_21days | I | 83 | 1277.349 | 1300.000 | 900.000 | 1640.000 | 1190.000 | 1360.000 | 1060.000 | 1480.000 | 19463.6 | 139.5121 | 121.0404 | 164.6893 |
| m_28days | I | 83 | 2002.651 | 2000.000 | 1300.000 | 2575.000 | 1830.000 | 2180.000 | 1670.000 | 2360.000 | 52605.1 | 229.3580 | 198.9906 | 270.7493 |
| m_34days | I | 83 | 2588.675 | 2620.000 | 1280.000 | 3400.000 | 2380.000 | 2840.000 | 2040.000 | 3140.000 | 121572.6 | 348.6726 | 302.5078 | 411.5963 |
| m_0days | II | 84 | 46.122 | 46.310 | 36.790 | 57.560 | 43.410 | 48.125 | 41.130 | 52.180 | 12.5 | 3.5309 | 3.0659 | 4.1636 |
| m_7days | II | 84 | 232.976 | 235.000 | 165.000 | 280.000 | 215.000 | 250.000 | 195.000 | 275.000 | 596.5 | 24.4225 | 21.2058 | 28.7986 |
| m_14days | II | 84 | 646.488 | 652.500 | 440.000 | 830.000 | 597.500 | 700.000 | 510.000 | 765.000 | 6306.5 | 79.4134 | 68.9539 | 93.6431 |
| m_21days | II | 84 | 1305.536 | 1300.000 | 820.000 | 1700.000 | 1200.000 | 1420.000 | 1070.000 | 1560.000 | 28551.8 | 168.9728 | 146.7174 | 199.2502 |
| m_28days | II | 84 | 1999.762 | 2000.000 | 1320.000 | 2610.000 | 1830.000 | 2170.000 | 1600.000 | 2450.000 | 67949.3 | 260.6709 | 226.3379 | 307.3792 |
| m_34days | II | 84 | 2610.119 | 2590.000 | 1880.000 | 3500.000 | 2370.000 | 2820.000 | 2080.000 | 3200.000 | 112080.7 | 334.7846 | 290.6900 | 394.7729 |
| m_0days | III | 84 | 45.801 | 45.960 | 36.710 | 55.200 | 43.335 | 47.555 | 40.640 | 53.800 | 14.67 | 3.8299 | 3.3255 | 4.5162 |
| m_7days | III | 84 | 233.571 | 230.000 | 185.000 | 285.000 | 220.000 | 250.000 | 195.000 | 270.000 | 513.60 | 22.6627 | 19.6778 | 26.7235 |
| m_14days | III | 83 | 636.771 | 630.000 | 170.000 | 775.000 | 595.000 | 700.000 | 520.000 | 750.000 | 7392.06 | 85.9771 | 74.5936 | 101.4930 |
| m_21days | III | 82 | 1299.573 | 1300.000 | 970.000 | 1545.000 | 1200.000 | 1400.000 | 1070.000 | 1500.000 | 19145.80 | 138.3684 | 119.9509 | 163.5201 |
| m_28days | III | 82 | 1975.524 | 1975.000 | 1430.000 | 2460.000 | 1790.000 | 2150.000 | 1660.000 | 2300.000 | 48042.05 | 219.1850 | 190.0104 | 259.0271 |
| m_34days | III | 82 | 2630.024 | 2640.000 | 1840.000 | 3200.000 | 2360.000 | 2900.000 | 2180.000 | 3080.000 | 94244.00 | 306.9919 | 266.1297 | 362.7950 |
| m_0days | IV | 84 | 45.577 | 45.235 | 37.300 | 55.310 | 43.295 | 48.390 | 39.410 | 51.330 | 13.6 | 3.6941 | 3.2075 | 4.3560 |
| m_7days | IV | 84 | 232.024 | 230.000 | 170.000 | 310.000 | 220.000 | 245.000 | 195.000 | 265.000 | 563.3 | 23.7345 | 20.6084 | 27.9873 |
| m_14days | IV | 84 | 633.857 | 635.000 | 465.000 | 890.000 | 590.000 | 675.000 | 510.000 | 754.000 | 5710.6 | 75.5682 | 65.6151 | 89.1089 |
| m_21days | IV | 84 | 1283.631 | 1285.000 | 950.000 | 1810.000 | 1185.000 | 1400.000 | 1030.000 | 1500.000 | 25947.2 | 161.0813 | 139.8653 | 189.9447 |
| m_28days | IV | 82 | 1980.244 | 1945.000 | 1530.000 | 2830.000 | 1820.000 | 2170.000 | 1620.000 | 2400.000 | 56479.0 | 237.6530 | 206.0202 | 280.8522 |
| m_34days | IV | 81 | 2639.753 | 2580.000 | 2020.000 | 3780.000 | 2380.000 | 2880.000 | 2160.000 | 3320.000 | 126984.9 | 356.3495 | 308.6628 | 421.6003 |
| m_0days | V | 84 | 45.209 | 45.305 | 37.200 | 51.750 | 43.285 | 47.395 | 40.180 | 49.440 | 9.0 | 3.0030 | 2.6075 | 3.5411 |
| m_7days | V | 84 | 226.607 | 232.500 | 140.000 | 275.000 | 215.000 | 245.000 | 180.000 | 260.000 | 673.6 | 25.9536 | 22.5353 | 30.6041 |

| Variable | Group | N | Mean | Median | Minimum | Maximum | Lower Quartile | Upper Quartile | Percentile 5.0 | Percentile 95.0 | Variance | Std. Dev. | Confidence SD -95.000% | Confidence SD +95.000% |
|---|---|---|---|---|---|---|---|---|---|---|---|---|---|---|
| m_14days | V | 83 | 629.699 | 640.000 | 430.000 | 880.000 | 575.000 | 690.000 | 450.000 | 755.000 | 7571.6 | 87.0147 | 75.4938 | 102.7179 |
| m_21days | V | 83 | 1272.530 | 1270.000 | 930.000 | 1600.000 | 1180.000 | 1400.000 | 980.000 | 1500.000 | 24221.6 | 155.6328 | 135.0267 | 183.7193 |
| m_28days | V | 83 | 1967.711 | 2000.000 | 1000.000 | 2430.000 | 1800.000 | 2180.000 | 1500.000 | 2400.000 | 74356.9 | 272.6846 | 236.5807 | 321.8950 |
| m_34days | V | 82 | 2602.073 | 2620.000 | 1480.000 | 3340.000 | 2400.000 | 2820.000 | 2080.000 | 3140.000 | 123912.9 | 352.0127 | 305.1581 | 415.9995 |
