# Supplementary material for: From In Vitro Screening to In Vivo Evaluation of Essential-Oil-Based Formulation Against Avian Pathogenic Escherichia coli
Source: Animals (Basel). 2026 Oct 2;16(19):3099. doi: 10.3390/ani16193099 (PMC13663412; doi:10.3390/ani16193099)
Supplement: Supplementary file 1 [file animals-16-03099-s001.zip › S8. Descriptive statistics for feed intake across experimental groups and measurement perio.pdf]

Supplementary Table S8. Descriptive statistics for feed intake across experimental groups and measurement periods.

Variable	Group	N	Mean	Median	Minimum	Maximum	Lower Quartile	Upper Quartile	Percentile 5.0	Percentile 95.0	Variance	Std. Dev.	Confidence SD -95.000%	Confidence SD +95.000%
0-7 days	I	4	3582.50	3615.00	3440.00	3660.00	3525.00	3640.00	3440.00	3660.00	9491.7	97.4252	55.1903	363.254
7-14 days	I	4	8523.75	8530.00	8280.00	8755.00	8352.50	8695.00	8280.00	8755.00	45006.3	212.1468	120.1789	790.999
14-21 days	I	4	14085.00	14135.00	13430.00	14640.00	13567.50	14602.50	13430.00	14640.00	370616.7	608.7829	344.8692	2269.876
21-28 days	I	4	20567.50	20635.00	19915.00	21085.00	20172.50	20962.50	19915.00	21085.00	262241.7	512.0954	290.0967	1909.372
28-34 days	I	4	20548.75	20522.50	20265.00	20885.00	20282.50	20815.00	20265.00	20885.00	97989.6	313.0329	177.3299	1167.158
0-7 days	II	4	3675.00	3685.00	3610.00	3720.00	3635.00	3715.00	3610.00	3720.00	2566.67	50.6623	28.69965	188.8967
7-14 days	II	4	8908.75	8895.00	8860.00	8985.00	8862.50	8955.00	8860.00	8985.00	3456.25	58.7899	33.30385	219.2008
14-21 days	II	4	14451.25	14417.50	14345.00	14625.00	14352.50	14550.00	14345.00	14625.00	16789.58	129.5746	73.40267	483.1251
21-28 days	II	4	20888.75	20935.00	20695.00	20990.00	20805.00	20972.50	20695.00	20990.00	17622.92	132.7513	75.20224	494.9696
28-34 days	II	4	20526.25	20517.50	20340.00	20730.00	20390.00	20662.50	20340.00	20730.00	29456.25	171.6282	97.22560	639.9240
0-7 days	III	4	3665.00	3640.00	3595.00	3785.00	3602.50	3727.50	3595.00	3785.00	7450	86.313	48.8956	321.824
7-14 days	III	4	8698.75	8760.00	8425.00	8850.00	8577.50	8820.00	8425.00	8850.00	35706	188.961	107.0444	704.550
14-21 days	III	4	14042.50	14307.50	13085.00	14470.00	13672.50	14412.50	13085.00	14470.00	414842	644.082	364.8657	2401.490
21-28 days	III	4	20618.75	20450.00	20225.00	21350.00	20332.50	20905.00	20225.00	21350.00	248973	498.972	282.6623	1860.440
28-34 days	III	4	20432.50	20605.00	18845.00	21675.00	19192.50	21672.50	18845.00	21675.00	2130642	1459.672	826.8888	5442.455
0-7 days	IV	4	3681.25	3650.00	3550.00	3875.00	3555.00	3807.50	3550.00	3875.00	24306	155.905	88.3183	581.298
7-14 days	IV	4	8678.75	8627.50	8330.00	9130.00	8422.50	8935.00	8330.00	9130.00	118606	344.393	195.0948	1284.084
14-21 days	IV	4	14100.00	13955.00	13415.00	15075.00	13490.00	14710.00	13415.00	15075.00	588700	767.268	434.6492	2860.795
21-28 days	IV	4	20595.00	20587.50	20465.00	20740.00	20507.50	20682.50	20465.00	20740.00	13617	116.690	66.1039	435.086
28-34 days	IV	4	20218.75	20210.00	18945.00	21510.00	19337.50	21100.00	18945.00	21510.00	1250240	1118.141	633.4153	4169.042
0-7 days	V	4	3596.25	3595.00	3490.00	3705.00	3517.50	3675.00	3490.00	3705.00	9373	96.814	54.8440	360.975
7-14 days	V	4	8670.00	8687.50	8445.00	8860.00	8540.00	8800.00	8445.00	8860.00	30950	175.926	99.6603	655.949
14-21 days	V	4	13887.50	13770.00	13580.00	14430.00	13630.00	14145.00	13580.00	14430.00	144225	379.770	215.1355	1415.989
21-28 days	V	4	20605.00	20867.50	19135.00	21550.00	19780.00	21430.00	19135.00	21550.00	1194450	1092.909	619.1215	4074.963
28-34 days	V	4	20413.75	20345.00	19425.00	21540.00	19630.00	21197.50	19425.00	21540.00	925240	961.894	544.9028	3586.466
