# Supplementary material for: From In Vitro Screening to In Vivo Evaluation of Essential-Oil-Based Formulation Against Avian Pathogenic Escherichia coli
Source: Animals (Basel). 2026 Oct 2;16(19):3099. doi: 10.3390/ani16193099 (PMC13663412; doi:10.3390/ani16193099)
Supplement: Supplementary file 1 [file animals-16-03099-s001.zip › S9. Weekly and cumulative feed conversion ratio across experimental groups of broilers..pdf]

Supplementary Table S9. Weekly and cumulative feed conversion ratio across experimental groups of broilers

Variable	Group	N	Mean	Median	Minimum	Maximum	Lower Quartile	Upper Quartile	Percentile 5.0	Percentile 95.0	Variance	Std. Dev.	Confidence SD -95.000%	Confidence SD +95.000%
0-7 days	I	4	1.004031	1.010341	0.973534	1.021906	0.989772	1.018289	0.973534	1.021906	0.000456	0.021344	0.012091	0.079583
7-14 days	I	4	1.039167	1.031967	1.010532	1.082200	1.019927	1.058406	1.010532	1.082200	0.000930	0.030493	0.017274	0.113695
14-21 days	I	4	1.024575	1.026849	0.984202	1.060403	1.002643	1.046508	0.984202	1.060403	0.000997	0.031572	0.017885	0.117718
21-28 days	I	4	1.366737	1.362199	1.348960	1.393589	1.350664	1.382809	1.348960	1.393589	0.000424	0.020587	0.011662	0.076760
28-34 days	I	4	1.692090	1.690194	1.618806	1.769166	1.630600	1.753579	1.618806	1.769166	0.005296	0.072773	0.041225	0.271339
0-34 days	I	4	1.275883	1.276228	1.264424	1.286654	1.269778	1.281988	1.264424	1.286654	0.000083	0.009128	0.005171	0.034033
0-7 days	II	4	0.936809	0.941690	0.912985	0.950871	0.923886	0.949732	0.912985	0.950871	0.000303	0.017400	0.009857	0.064877
7-14 days	II	4	1.026116	1.024379	1.009045	1.046663	1.014591	1.037642	1.009045	1.046663	0.000252	0.015870	0.008990	0.059173
14-21 days	II	4	1.044810	1.043648	1.013865	1.078078	1.027803	1.061816	1.013865	1.078078	0.000691	0.026295	0.014896	0.098043
21-28 days	II	4	1.434237	1.420741	1.392013	1.503451	1.401316	1.467157	1.392013	1.503451	0.002381	0.048794	0.027641	0.181931
28-34 days	II	4	1.601708	1.608068	1.568688	1.622010	1.583496	1.619921	1.568688	1.622010	0.000591	0.024318	0.013776	0.090670
0-34 days	II	4	1.271463	1.277425	1.247303	1.283696	1.260537	1.282389	1.247303	1.283696	0.000277	0.016645	0.009429	0.062062
0-7 days	III	4	0.929757	0.930573	0.912829	0.945053	0.918480	0.941034	0.912829	0.945053	0.000202	0.014200	0.008044	0.052944
7-14 days	III	4	1.048659	1.030700	1.026869	1.106369	1.026954	1.070365	1.026869	1.106369	0.001492	0.038631	0.021884	0.144038
14-21 days	III	4	1.045658	1.042931	1.025899	1.070869	1.027702	1.063614	1.025899	1.070869	0.000467	0.021614	0.012244	0.080588
21-28 days	III	4	1.494048	1.444556	1.430677	1.656402	1.431521	1.556574	1.430677	1.656402	0.011857	0.108890	0.061685	0.406000
28-34 days	III	4	1.531762	1.574958	1.375754	1.601377	1.469179	1.594345	1.375754	1.601377	0.011074	0.105233	0.059613	0.392366
0-34 days	III	4	1.274071	1.270494	1.269601	1.285694	1.269652	1.278490	1.269601	1.285694	0.000061	0.007787	0.004411	0.029035
0-7 days	IV	4	0.939906	0.937574	0.923411	0.961064	0.927136	0.952675	0.923411	0.961064	0.000274	0.016540	0.009370	0.061671
7-14 days	IV	4	1.028437	1.028751	1.018337	1.037908	1.022682	1.034191	1.018337	1.037908	0.000066	0.008121	0.004600	0.030279
14-21 days	IV	4	1.033396	1.035045	1.018699	1.044792	1.024030	1.042761	1.018699	1.044792	0.000139	0.011775	0.006670	0.043902
21-28 days	IV	4	1.512963	1.506889	1.441094	1.596980	1.444203	1.581723	1.441094	1.596980	0.006466	0.080408	0.045551	0.299806
28-34 days	IV	4	1.592591	1.533445	1.453378	1.850098	1.470934	1.714249	1.453378	1.850098	0.032243	0.179563	0.101720	0.669508
0-34 days	IV	4	1.282518	1.285451	1.246547	1.312623	1.261658	1.303378	1.246547	1.312623	0.000789	0.028096	0.015916	0.104757
0-7 days	V	4	0.943942	0.943900	0.933008	0.954958	0.937095	0.950788	0.933008	0.954958	0.000085	0.009232	0.005230	0.034422

Variable	Group	N	Mean	Median	Minimum	Maximum	Lower Quartile	Upper Quartile	Percentile 5.0	Percentile 95.0	Variance	Std. Dev.	Confidence SD -95.000%	Confidence SD +95.000%
7-14 days	V	4	1.044609	1.043197	1.008656	1.083387	1.021084	1.068134	1.008656	1.083387	0.000996	0.031559	0.017878	0.117670
14-21 days	V	4	1.041125	1.044236	1.017610	1.058419	1.026398	1.055852	1.017610	1.058419	0.000345	0.018576	0.010523	0.069261
21-28 days	V	4	1.429678	1.432791	1.339048	1.514085	1.373289	1.486068	1.339048	1.514085	0.005545	0.074462	0.042182	0.277635
28-34 days	V	4	1.636287	1.638484	1.559739	1.708441	1.575255	1.697319	1.559739	1.708441	0.005210	0.072177	0.040888	0.269116
0-34 days	V	4	1.282644	1.285875	1.248660	1.310167	1.259923	1.305365	1.248660	1.310167	0.000788	0.028076	0.015905	0.104683
